# Ancestry inference using principal component analysis and spatial analysis: a distance-based analysis to account for population substructure

**DOI:** 10.1186/s12864-017-4166-8

**Published:** 2017-10-16

**Authors:** Jinyoung Byun, Younghun Han, Ivan P. Gorlov, Jonathan A. Busam, Michael F. Seldin, Christopher I. Amos

**Affiliations:** 1 Department of Biomedical Data Science, Dartmouth Geisel School of Medicine, One Medical Center Drive, Lebanon, NH 03756 USA; 2 Department of Biochemistry and Molecular Medicine, University of California Davis, Davis, CA 95616 USA

**Keywords:** Ancestry inference, Principal component analysis, Spatial analysis, Inverse distance weighted interpolation

## Abstract

**Background:**

Accurate inference of genetic ancestry is of fundamental interest to many biomedical, forensic, and anthropological research areas. Genetic ancestry memberships may relate to genetic disease risks. In a genome association study, failing to account for differences in genetic ancestry between cases and controls may also lead to false-positive results. Although a number of strategies for inferring and taking into account the confounding effects of genetic ancestry are available, applying them to large studies (tens thousands samples) is challenging. The goal of this study is to develop an approach for inferring genetic ancestry of samples with unknown ancestry among closely related populations and to provide accurate estimates of ancestry for application to large-scale studies.

**Methods:**

In this study we developed a novel distance-based approach, Ancestry Inference using Principal component analysis and Spatial analysis (AIPS) that incorporates an Inverse Distance Weighted (IDW) interpolation method from spatial analysis to assign individuals to population memberships.

**Results:**

We demonstrate the benefits of AIPS in analyzing population substructure, specifically related to the four most commonly used tools EIGENSTRAT, STRUCTURE, fastSTRUCTURE, and ADMIXTURE using genotype data from various intra-European panels and European-Americans. While the aforementioned commonly used tools performed poorly in inferring ancestry from a large number of subpopulations, AIPS accurately distinguished variations between and within subpopulations.

**Conclusions:**

Our results show that AIPS can be applied to large-scale data sets to discriminate the modest variability among intra-continental populations as well as for characterizing inter-continental variation. The method we developed will protect against spurious associations when mapping the genetic basis of a disease. Our approach is more accurate and computationally efficient method for inferring genetic ancestry in the large-scale genetic studies.

**Electronic supplementary material:**

The online version of this article (10.1186/s12864-017-4166-8) contains supplementary material, which is available to authorized users.

## Background

During the last decade, genome-wide association studies (GWAS) have helped identify a large number of allelic variants for common complex traits and diseases. Because many of the associations from these studies show small to modest effects in nature with a very strict alpha-level of statistical significance, robust conclusions from them require careful analysis to exclude false-positive results. Population stratification, the presence of systematic allele frequency differences between populations or subpopulations, can cause spurious associations and distortions in effect estimates between genetic variants and disease [1–5]. Closely related individuals may have a more similar disease risk than distantly related individuals. This risk homogeneity among individuals of similar ancestries may result from lifestyle similarities or the presence of one or more risk-conferring alleles [5]. However, several alleles may differ between ancestry groups that do not confer risk. Thus, some level of correlation with shared ancestry in GWAS can introduce bias leading to excess false-positives unless a proper correction of population stratification is performed [2]. To detect whether there is confounding due to population stratification, genomic control and structured association applications are used. Several publications have described the selection of ancestry informative markers (AIMs), used to infer genetic ancestry [4, 6–13]. Basing analysis on AIMs rather than all markers that might have been analyzed in a GWAS allows a more parsimonious use of the data and the markers are typically selected to avoid strong linkage disequilibrium among the markers.

There are two commonly used types of analytical approaches to describe genetic similarities: distance-based and model-based approaches. The *distance-based approach* adopts a pairwise distance matrix computed among each pair of individuals and the *model-based approach* uses parametric models such as maximum-likelihood or Bayesian methods.

Menozzi et al. constructed synthetic maps of human gene frequencies in Europeans using genetic distance among population pairs [14]. They used principal component analysis (PCA) to generate a single geographic map from individual allele frequencies. The most commonly used software packages for accurately analyzing admixture population structures are EIGENSTRAT [15, 16], STRUCTURE [17] and fastStructure [18]. Price et al. developed EIGENSTRAT to detect and correct for population stratification using principal component analysis (PCA) of genotyped data to extract linear combinations of individuals that share the greatest similarities. EIGENSTRAT calculates the pattern of individual similarity in relation to markers. In the case of data with very large numbers of individuals in relation to markers, it is computationally demanding to compute the eigenvectors. Also, this does not provide any inference of ancestry membership. Pritchard introduced STRUCTURE, a Bayesian model-based clustering method, to estimate population structure and assign individuals into population membership groups based on their genotypes under the assumption that the marker loci are unlinked and at linkage equilibrium with one another within populations [17]. With STRUCTURE, a variational Bayesian inference method was applied to compute approximate ancestry inference using the log-marginal likelihood of the data by proposing a family of tractable parametric posterior distributions over the hidden variables in the model. Inferring population structures in larger data sets with this method is computationally challenging because it requires intensive computation time and resources and may have convergence problems in fitting Markov Chain Monte Carlo based posterior samplings. In 2014, Raj proposed fastSTRUCTURE to reduce the computational time and complexity while attempting to achieve accuracy comparable to STRUCTURE [18]. ADMIXTURE is an additional popular program and uses a likelihood-based approach [19, 20].

A distance-based approach such as multidimensional scaling could also be applied, but the groups identified from evaluating a pairwise distance (similarity) matrix may be heavily dependent on both the distance measure and the graphical representation. A challenge in large-scale genetic studies is to understand the underlying data structure so as to identify whether individuals are from a homogeneous population or from heterogeneous subpopulations. When samples become larger and detected effects of genetic loci on disease phenotype become smaller, confounding with ancestry may introduce a greater number of false-positive results. Guan et al. proposed a genetic similarity score matching method (GSM) to correct population stratification using individual-based matching [21]. GSM matches case-control subjects based on the average proportion of alleles using identity-by-state (IBS) measures that indicate the degree of similarity over tens of thousands of SNPs. A different approach was taken by Lee et al. who developed a variation of genetic matching (GEM) called Spectral-GEM that replaces the PCA used in GEM with significant ancestry components derived from the spectral graph theory [22].

More recently, Li et al. introduced an algorithmic approach, FastPop to infer the ancestry membership for the intercontinental study [23]. It is a distance-based method that reflects the clines of intermarriage among continental groups using a triangle connecting the known ancestry centroids. It could be easily applied to three or four intercontinental origins using triangle or tetrahedron shapes, respectively.

In this study we introduce a novel distance-based inference of ancestry membership with commonly used ancestry informative markers (AIMs). This novel approach can accurately infer ancestry memberships from a pairwise distance matrix calculated between individuals and centroids of the known populations using HapMap or Human Genome Diversity Project (HGDP) samples. The main aim of this method is to identify the unrevealed sub-structures and to infer the correct inference of ancestry memberships for samples with unknown ethnicity.

## Methods

### Principal component analysis

Principal component analysis (PCA) is one of the most useful statistical tools for analyzing multivariate data and has been widely applied to high-dimensional genetics or genomics data. PCA uses spectral (eigenvalue) decomposition to transform a number of correlated variables into a smaller number of uncorrelated variables, which are called principal components (PCs) with a minimum loss of information. The reduced numbers of top ranked PCs are calculated by projecting samples onto spaces spanned by the eigenvectors of the sample covariance matrix and selecting the eigenvectors that comprise the largest contribution of sample variation [24]. To perform PCA, there are two approaches using eigenvalue decomposition (P-mode) and singular value decomposition (Q-mode). The eigenvalue decomposition method uses the covariance relationships between markers and the singular value decomposition method uses covariance among individuals.

Initially genome wide association studies have a larger number of SNPs (***p***) compared to the size of samples (***n***), in which case principal components analysis is performed in the Q-mode and can be obtained by calculating the eigenvectors and eigenvalues of a covariance matrix whose rank is at most ***n*** **-1**. The axes of the eigenvectors with the largest eigenvalues are usually important in describing within-continent genetic variations and can correct for the confounding effects of population substructure. The eigenvectors so derived provide insights into variability among individuals but are specific to the specific population studied and cannot be applied to future populations. As N increases, the computational burden of computing the intraindividual correlation matrix increases exponentially. EIGENSTRAT was developed for analysis when the number of samples is far less than the number of markers, but more recent studies such as the Oncoarray [25] and the UK Biobank [26] present scenarios with very large sample sizes for which Q-mode analysis is not practical.

Eigenvectors between markers derived by P-mode in a population can be used as the SNP-weights (loadings) that enable researchers in a large consortium to compute the new variance components (scores) in new data with the nature of the similarity in the markers. The SNP-weights so derived can help reduce the time to compute principal components by omitting the computational step of deriving the correlation matrix in markers with a specified AIMs. Because only a selected set of markers are informative about the population substructure, the number of markers that need to be included in a P-mode analysis can be limited to under ~25,000, which is computationally feasible for deriving correlation structures (Additional file 1: Supplementary Methods).

### Spatial analysis; inverse distance weighted interpolation approach

Spatial analysis is used to manipulate spatial information to extract distance relationship information. Spatial interpolation is the application of spatial analysis to estimate values at unknown points with known values. As a common example, to predict precipitation in a certain area when not given entire weather information, spatial interpolation enables one to estimate precipitation in locations without recorded data using known weather information. In the Inverse Distance Weighted (IDW) interpolation method, the sample points are weighted during interpolation such that the influence of one point relative to another decreases with distance from the unknown point.

### Ancestry inference using PCA scores and spatial interpolation

IDW interpolation assumes that points that are close to one another are more alike than those that are farther apart. To infer ancestry membership proportion for an unknown sample, IDW computes the distance metrics from each centroid of each known population. Those estimated values closest to the centroid of a known population will be assigned a higher proportion of ancestry that diminishes with distance and will be weighted greater than those populations that are farther away. To identify centroids of known populations, we obtained data from samples that had known European ancestry, as further described in the results.

**Fig. 1:**
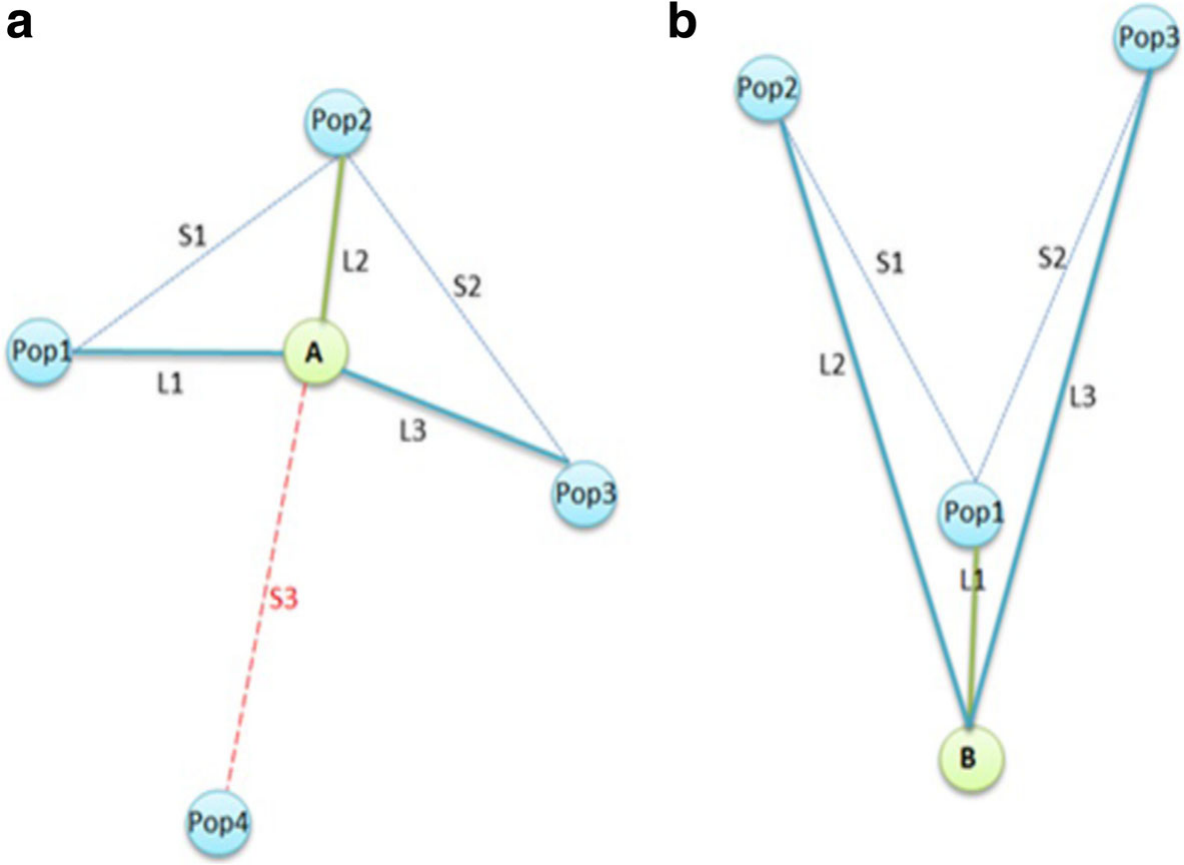
**a** Selection of Admixtures. In a model with 3 admixtures, L2 is the shortest distance between sample A and a centroid of known population (Pop2). Then compare two other closest populations; Pop1 and Pop3 with the distances, S1 and S2, between the closest Pop2 and two other closer ones; Pop1 and Pop3. If S1 and S2 are longer than L1 and L3, respectively, then keep Pop1 and Pop3 in the 3 admixture model. Pop4 has longer distance than other three populations then the Pop4 is not included. **b** After selecting the closest population (Pop1) to sample B, compare two other closest populations (Pop2 and Pop3). In this case, S1 and S2 are shorter than L2 and L3. Then Pop2 and Pop3 would not be included in the 3 admixture model

For admixture membership, we sorted all distances among individuals by each population centroid, chose the number of admixtures denoted by *s*, and then found the first *s* closest population centroids to each individual. We then computed the distances from the centroid of the closest population to the centroids of other nearby populations. Next, we compared the distance from the second closest population centroid to each individual in relation to the distance between the two closest population centroids to each other. If the distance between the two closest population centroids was longer than the distance between the individual and the second closest population centroid, the second closest population was considered in admixture model, and so on as shown in Fig. 1.

Inverse distance weighted (IDW) interpolation in spatial analysis was then used to infer individual genetic ancestry. We applied two different spatial weights based on the centroid distances: power-distance (PD) weights and exponential-distance (ED) weights. Formally, let ***x***_***ik***_ be the kth score for the ith individual, ***x***_***jk***_ the kth centroid in the jth subpopulation. The power-distance weights function, ${\Delta}_{{\boldsymbol{p}}_{\boldsymbol{ijk}}}^{\boldsymbol{PD}}$ is a negative power function of distance given by,

$$ {\Delta}_{{\boldsymbol{p}}_{\boldsymbol{ijk}}}^{\boldsymbol{PD}}=\frac{{\boldsymbol{PD}}_{\boldsymbol{ijk}}^{-\boldsymbol{\alpha}}}{\sum_{\boldsymbol{j}=1}^{\#.\boldsymbol{pop}}{\boldsymbol{PD}}_{\boldsymbol{ijk}}^{-\boldsymbol{\alpha}}}\ \mathrm{and}\ {\boldsymbol{PD}}_{\boldsymbol{ijk}}=\sqrt{\sum_{\boldsymbol{k}=1}^{\#.\boldsymbol{Scores}}{\left({\boldsymbol{x}}_{\boldsymbol{ik}}-{\boldsymbol{x}}_{\boldsymbol{j}\boldsymbol{k}}\right)}^2}. $$

The exponential-distance weights function, ${\Delta }_{{\boldsymbol{p}}_{\boldsymbol{ijk}}}^{\boldsymbol{ED}}$ with the negative exponential function is given by,

$$ {\Delta}_{{\boldsymbol{p}}_{\boldsymbol{ijk}}}^{\boldsymbol{ED}}=\frac{{\boldsymbol{e}}^{-\boldsymbol{\alpha} \cdot {\boldsymbol{ED}}_{\boldsymbol{ijk}}}}{\sum_{\boldsymbol{j}=1}^{\#.\boldsymbol{pop}}{\boldsymbol{e}}^{-\boldsymbol{\alpha} \cdot {\boldsymbol{ED}}_{\boldsymbol{ijk}}}}\ \mathrm{and}\ {\boldsymbol{ED}}_{\boldsymbol{ijk}}=\sqrt{\sum_{\boldsymbol{k}=1}^{\#.\boldsymbol{Scores}}{\left({\boldsymbol{x}}_{\boldsymbol{ik}}-{\boldsymbol{x}}_{\boldsymbol{j}\boldsymbol{k}}\right)}^2}. $$

We developed a novel approach where eigenvalues contribute additional weights. The size of eigenvalues reflects the proportion of total variance explained by the eigenvector and larger eigenvalues should be upweighted to allow for greater variance compared to smaller eigenvalues. Formally, an ancestry inference, ${\Delta}_{{\boldsymbol{p}}_{\boldsymbol{ijk}}}^{\boldsymbol{EVD}}$ is computed and normalized by the inverse distance weighted on each eigenvalue:

$$ {\Delta}_{{\boldsymbol{p}}_{\boldsymbol{ijk}}}^{\boldsymbol{EV}\boldsymbol{D}}=\frac{{\boldsymbol{EV}\boldsymbol{D}}_{\boldsymbol{ijk}}^{-\boldsymbol{\alpha}}}{\sum_{\boldsymbol{j}=1}^{\#.\boldsymbol{pop}}{\boldsymbol{EV}\boldsymbol{D}}_{\boldsymbol{ijk}}^{-\boldsymbol{\alpha}}}\kern0.5em \mathrm{and}\ {\boldsymbol{EV}\boldsymbol{D}}_{\boldsymbol{ijk}}=\sqrt{\sum_{\boldsymbol{k}=1}^{\#.\boldsymbol{Scores}}{\left({\boldsymbol{x}}_{\boldsymbol{ik}}-{\boldsymbol{x}}_{\boldsymbol{j}\boldsymbol{k}}\right)}^2\cdot {\boldsymbol{EV}}_{\boldsymbol{k}}/{\sum}_{\boldsymbol{k}=1}^{\#.\boldsymbol{Scores}}{\boldsymbol{EV}}_k}, $$

where ***EV***_***k***_ is the eigenvalue of kth score and ***EVD***_***ijk***_ is the weighted distance from each centroid of the known subpopulation to an individual. When we add eigenvalues as weights for inferring ancestry origin, the larger eigenvalue that has more weight can reveal which cluster may be closer and more appropriate to each individual (Additional file 2: Figure S1).

To utilize this methodology, we created the R-package AIPS that allows one to calculate SNP weights and scores from PCA, predict scores from SNP weights computed on the same pre-defined AIMs and infer genetic ancestry using pre-defined ancestry clustering information. AIPS can be performed on samples larger than markers and vice versa. After generating a matrix of SNP weights from large enough samples of AIMs, AIPS predicts a score matrix projected from the largest variance components. For missing genotype values, it computes the mean SNP value and replaces a missing genotype value with the mean SNP value. The eigenvectors and eigenvalues were calculated from correlation matrix based on standardizing each SNP column with zero mean and unit standard deviation.

## Results

### Application in European subpopulations and European AIMs

**Fig. 2:**
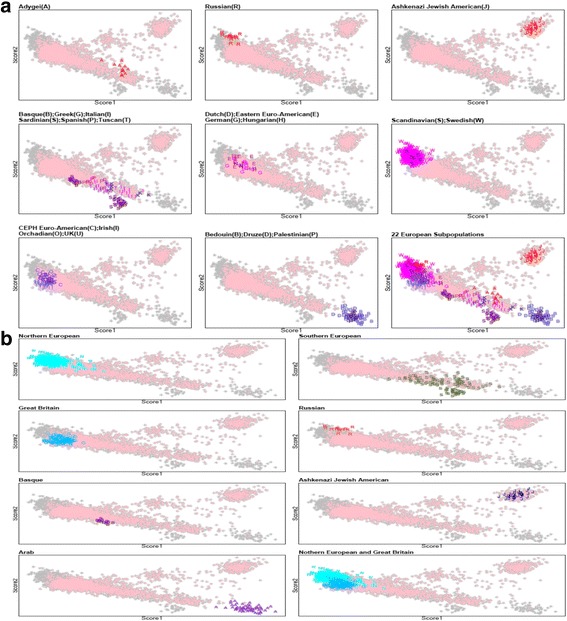
**a** Population structure within Europe using 22 diverse sets of European descendants. The scores were calculated by AIPS. The colored points in grey and pink indicate all 4376 Europeans and 3424 individuals with unknown ancestry memberships in subpopulations, respectively. 952 known ancestry individuals in 22 subpopulations were overplotted on all 4376 Europeans. **b** European substructure analysis using scores from Principal Component Analysis. Among 952 ancestry known individuals, 7 subgroups within Europe were defined; Northern European group, Southern European group, Great Britain, Russian, Basque, Ashkenazi Jewish American, and Arab group. For Northern European group, Dutch American, Eastern European American, German American, Hungarian American, Scandinavian American, and Swedish were assigned. Southern European group consisted of Adygei, Greek American, Italian American, Sardinian, Spanish, and Tuscan. For Great Britain, CEPH Euro American, Irish, Orcadian, and United Kingdom American were assigned. Bedouin, Druze and Palestinian were defined as Arab group

To demonstrate the application of AIPS, we performed an intra-European analysis involving 4376 individuals of European descent with a set of 25,732 pre-selected known Intra-European AIMs. For European genetic substructure studies presented in Fig. 2 (a) and Additional file 2: Table S1, we used data from the Human Genome Diversity Panel (HGDP), HapMap, Italian, Spanish, Swedish, and European Americans along with subpopulation unknown individuals from the New York Cancer Project and the Children’s Hospital of Philadelphia from the Illumina-control database (I-ControlDB). The approach to selecting subpopulations for characterizing European ancestry has been previously presented [13]. Of the 4376 individuals, 3424 participants from the New York Cancer Project and the Children’s Hospital of Philadelphia were self-identified as Europeans and had unknown subpopulation ancestry among intra-European and closely related population clusters, while 952 individuals from 22 ancestry-known subpopulations (Adygei, Ashkenazi Jewish American, Basque, Bedouin, Druze, Palestine, CEPH European American, Eastern European American, German American, Greek American, Hungarian American, Irish, Italian American, Tuscan, Netherland, Orcadian, Russian, Sardinian, Scandinavian, Swedish, Spanish, United Kingdom American) were chosen to compute centroids from each European subpopulation as the known ancestry clusters [20]. In addition, we also collapsed 22 subpopulations into 7 major ethnic groups of Europeans, Arab, and Jews based on geographical and genetic differences (Fig. 2 b). To clarify the genetic heterogeneity among 7 major ethnic groups consisting of Northern European, Southern European, Great Britain, Russian, Basque, Arab, and Jew, we performed Hotelling’s T^2^ tests among 7 different centroids of subpopulations, as presented in Table 1.

**Table 1:** Comparison among 7 subpopulations within Europe using Hotelling’s T^2^ test

Population1	Population2	Statistic	*P*-value	*P*-value*
N. European	S. European	334.97	< 1 × 10^−16^	< 1 × 10^−4^
N. European	Great Britain	331.63	< 1 × 10^−16^	< 1 × 10^−4^
N. European	Russian	148.56	< 1 × 10^−16^	< 1 × 10^−4^
N. European	Arab	81.87	1.12 × 10^−14^	< 1 × 10^−4^
N. European	Basque	181.06	< 1 × 10^−16^	< 1 × 10^−4^
N. European	Jews	362.28	< 1 × 10^−16^	< 1 × 10^−4^
S. European	Great Britain	680.60	< 1 × 10^−16^	< 1 × 10^−4^
S. European	Russian	713.40	< 1 × 10^−16^	< 1 × 10^−4^
S. European	Arab	334.90	< 1 × 10^−16^	< 1 × 10^−4^
S. European	Basque	710.36	< 1 × 10^−16^	< 1 × 10^−4^
S. European	Jews	1108.18	< 1 × 10^−16^	< 1 × 10^−4^
Great Britain	Russian	865.25	< 1 × 10^−16^	< 1 × 10^−4^
Great Britain	Arab	646.45	< 1 × 10^−16^	< 1 × 10^−4^
Great Britain	Basque	1165.79	< 1 × 10^−16^	< 1 × 10^−4^
Great Britain	Jews	73.14	7.77 × 10^−15^	< 1 × 10^−4^
Russian	Arab	17.64	1.04 × 10^−8^	1 × 10^−4^
Russian	Basque	4.96	0.0014	0.0014
Russian	Jews	1436.50	< 1 × 10^−16^	< 1 × 10^−4^
Arab	Basque	16.82	2.34 × 10^−8^	< 1 × 10^−4^
Arab	Jews	1038.41	< 1 × 10^−16^	< 1 × 10^−4^
Basque	Jews	1366.32	< 1 × 10^−16^	< 1 × 10^−4^

*P*-value* is computed using permutation test which estimates the non-parametric *P*-value for the hypothesis test in Hotelling’s T^2^ test

We compared eigenvalues and principal components between AIPS and EIGENSTRAT. The correlation rates of eigenvalues and eigenvectors between AIPS and EIGENSTRAT are close to 1 even though the PC scales between the two approaches, scores projected from SNP weights and eigenvectors as the pattern of individual dissimilarity are different (Additional file 2: Figure S2). For the initial assessment, 952 individuals in either 22 or 7 collapsed Euro-subpopulations were selected to compare the proportions of ancestry population memberships among AIPS, STRUCTURE and fastSTRUCTURE. These samples and AIMs have been analyzed in many population studies [1, 4, 19, 20, 27]. Since the ancestry memberships for 952 samples are known, it is easy to identify the ethnic agreement between each individual and subpopulation cluster. STRUCTURE using Bayesian methods to differentiate population structures is feasible for limited sample sizes and small marker numbers [28]. Among 22 European and near Eastern subpopulations, AIPS performed better in inferring the proportions of ancestry memberships under the option in which each individual can be a descendent of between 2 and 4 populations (Additional file 2: Figure S3), while STRUCTURE with and without population labels and fastSTRUCTURE could not elucidate the heterogeneity and admixture among many of the populations in 22 clusters presented in Additional file 2: Table S1 (Additional file 2: Figure S4). In fastSTRUCTURE, we applied two types of priors; simple and logistic. fastSTRUCTURE could not recognize the differences between individuals of the 22 subpopulations. By default the number of eigenvalues in AIPS is five that are significant from the plot of the eigenvalues. AIPS allows one to have admixtures from up to number of populations. For 7 subpopulation study, we can assume at most 7 admixtures and AIPS computes 3 admixtures by default. AIPS using the top five ranked eigenvalues and the different number of admixtures displayed distinguishable population structures for inferring ethnic memberships whereas fastSTRUCTURE was unable to discriminate the ethnic heterogeneity among different population clusters.

We computed the pairwise difference of distances between centroids in two populations and ranked them based on the closeness among 22 European subpopulations (Additional file 2: Table S2 and S3). The ranks based on the geogenetic distances between them provide the clear interpretation between the geographical and population structures. We reduced the number of subpopulations using distance-based analysis and geographical relatedness. After grouping 22 subpopulations into geographically closer clusters based on PCA and distance-based analysis, we assigned 7 subpopulations: Northern European, Southern European, Great Britain, Russian, Arab, Basque, and Jews. To check whether 7 out of 22 subpopulations are substantially distinct from each other, we performed Hotelling’s T^2^ test, which compares the difference in two multivariate means. The reassigned clusters were clearly distinguishable in terms of genetic and geographical differences.

**Fig. 3:**
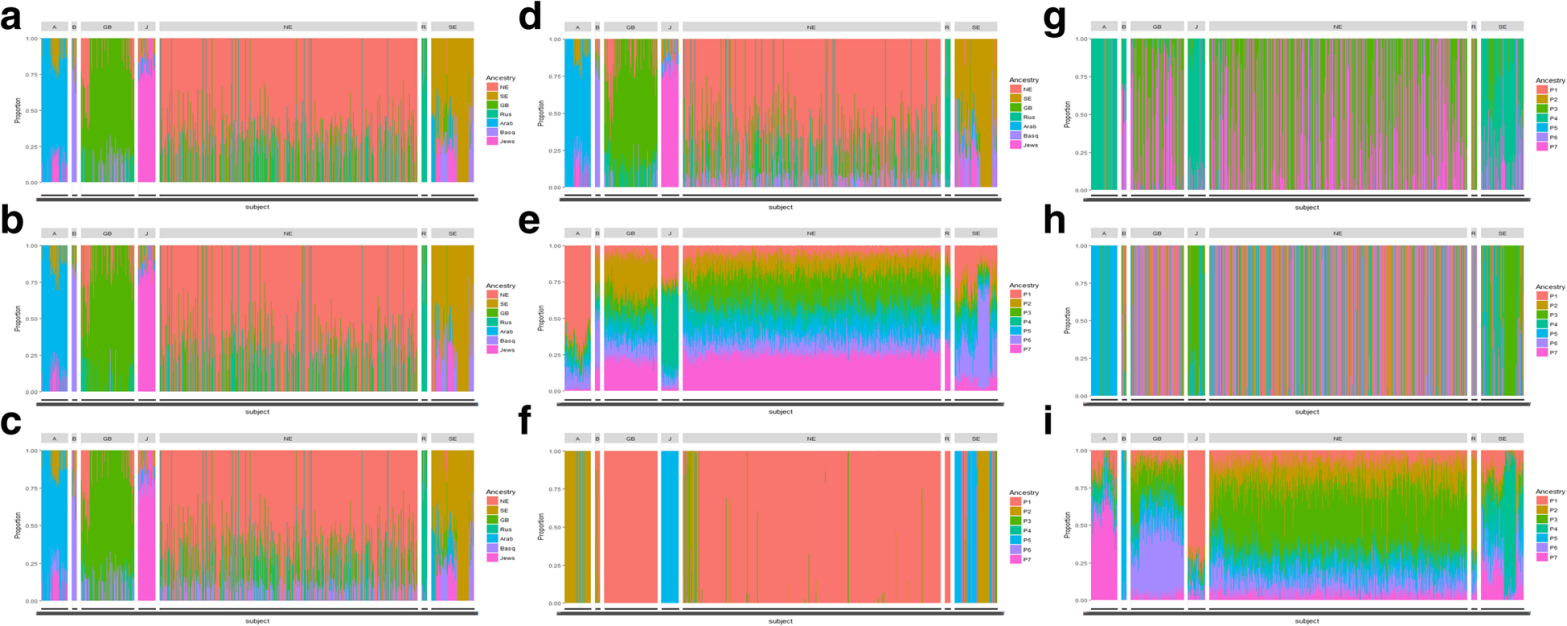
**a** AIPS assuming 3 admixtures using IDW; **b** AIPS assuming 3 admixtures using IDW with Eigenvalue Weight; **c** AIPS assuming 4 admixtures using IDW; **d** AIPS assuming 4 admixtures using IDW with Eigenvalue Weight; **e** Structure not given POPID; **f** Structure given POPID; **g** fastSTRUCTURE using option “simple”; **h** fastSTRUCTURE using option “logistic prior”; **i** ADMIXTURE without reference population information

To assess the accuracy level of inferring ethnic membership in a large number of populations, we reanalyzed 952 individuals with identified ethnicities using AIPS, STRUCTURE, fastSTRUCTURE, and ADMIXTURE among 7 collapsed subpopulations. As shown in Fig. 3 AIPS in the different number of admixtures represented clear discrimination in ancestry memberships among 7 different clusters while STRUCTURE and fastSTRUCTURE performed very poorly in calculating these ancestry inferences and distinguishing all seven clusters. The graphical figure from STRUCTURE without pre-specified Population IDs seemed to find largely homogeneous population structures; {Great Britain, Russian, Basque, and Northern European}; {Jews and Southern European}; and {Arab}, as further described in Table 2. To quantitate the average of accuracy in assigned groups, the average proportion for correct inference of each assigned ethnic group is computed using

$$ \boldsymbol{Avg}\%\boldsymbol{Correct}=\frac{\sum_{\boldsymbol{j}=\mathbf{1}}^{\boldsymbol{p}}\frac{\sum_{\boldsymbol{i}=\mathbf{1}}^{{\boldsymbol{n}}_{\boldsymbol{p}}}\boldsymbol{Avg}\%{\boldsymbol{Correct}}_{\boldsymbol{i}}}{{\boldsymbol{n}}_{\boldsymbol{p}}}}{\boldsymbol{p}}, $$

**Table 2:** The Average percent of correctly inferred proportions from AIPS, STRUCTURE, and ADMIXTURE

Given Pop	Inferred Clusters							Number of Individuals(n_p_)
AIPS[3]	NE^a^	SE^b^	GB^c^	Russia^d^	Arab^e^	Basque^f^	Jew^g^	
NE	*0.78*	0.00	0.11	0.11	0.00	0.00	0.00	601
SE	0.00	*0.68*	0.04	0.00	0.08	0.12	0.08	100
GB	0.11	0.00	*0.77*	0.05	0.00	0.07	0.00	124
Russia	0.05	0.00	0.08	*0.87*	0.00	0.00	0.00	13
Arab	0.00	0.08	0.00	0.00	*0.83*	0.00	0.09	62
Basque	0.00	0.08	0.05	0.00	0.00	*0.87*	0.00	12
Jew	0.00	0.06	0.00	0.00	0.04	0.01	*0.90*	40
AIPS[4]	NE^a^	SE^b^	GB^c^	Russia^d^	Arab^e^	Basque^f^	Jew^g^	n_p_
NE	*0.74*	0.00	0.10	0.11	0.00	0.05	0.00	601
SE	0.01	*0.65*	0.06	0.00	0.09	0.11	0.07	100
GB	0.11	0.00	*0.76*	0.05	0.00	0.09	0.00	124
Russia	0.05	0.00	0.08	*0.83*	0.00	0.04	0.00	13
Arab	0.00	0.07	0.00	0.00	*0.80*	0.05	0.08	62
Basque	0.04	0.08	0.05	0.00	0.00	*0.83*	0.00	12
Jew	0.00	0.05	0.00	0.00	0.04	0.04	*0.87*	40
STRUCTURE^1^	POP1	POP2	POP3	POP4	POP5	POP6	POP7	n_p_
NE	0.07	0.13	*0.21*	0.10	0.14	0.09	0.26	601
SE	0.21	0.09	0.05	0.10	0.14	0.33	0.07	100
GB	0.07	*0.28*	0.11	0.09	0.13	0.11	0.22	124
Russia	0.10	0.04	0.06	0.10	*0.34*	0.04	*0.33*	13
Arab	*0.64*	0.04	0.03	0.09	0.07	0.11	0.01	62
Basque	0.08	0.22	0.03	0.10	0.05	*0.35*	0.16	12
Jew	0.25	0.04	0.03	*0.54*	0.05	0.07	0.02	40
STRUCTURE^2^	POP1	POP2	POP3	POP4	POP5	POP6	POP7	n_p_
NE	*0.93*	0.03	0.03	0.00	0.00	0.00	0.00	601
SE	0.17	0.28	0.06	0.00	*0.46*	0.00	0.03	100
GB	*1.00*	0.00	0.00	0.00	0.00	0.00	0.00	124
Russia	*1.00*	0.00	0.00	0.00	0.00	0.00	0.00	13
Arab	0.00	*0.89*	0.00	0.00	0.11	0.00	0.00	62
Basque	*0.64*	0.27	0.09	0.00	0.00	0.00	0.00	12
Jew	0.00	0.00	0.00	0.00	*1.00*	0.00	0.00	40
ADMIXTURE^1^	POP1	POP2	POP3	POP4	POP5	POP6	POP7	n_p_
NE	0.07	0.18	*0.42*	0.06	0.11	0.11	0.05	601
SE	0.11	0.07	0.05	*0.37*	0.12	0.12	0.15	100
GB	0.06	0.07	0.22	0.06	0.15	*0.42*	0.04	124
Russia	0.04	*0.63*	0.05	0.03	0.10	0.12	0.03	13
Arab	0.14	0.03	0.04	0.12	0.06	0.05	*0.57*	62
Basque	0.00	0.02	0.01	0.03	*0.92*	0.01	0.00	12
Jew	*0.73*	0.03	0.03	0.04	0.09	0.04	0.04	40
AIPS[3]	NE^a^	SE^b^	GB^c^	Russia^d^	Arab^e^	Basque^f^	Jew^g^	n_p_
NE	*0.82*	0.00	0.09	0.08	0.00	0.01	0.00	601
SE	0.00	*0.69*	0.04	0.00	0.08	0.13	0.07	100
GB	0.12	0.00	*0.79*	0.02	0.00	0.07	0.00	124
Russia	0.07	0.00	0.05	*0.88*	0.00	0.00	0.00	13
Arab	0.00	0.07	0.00	0.00	*0.87*	0.00	0.07	62
Basque	0.02	0.05	0.06	0.00	0.00	*0.86*	0.00	12
Jew	0.00	0.05	0.00	0.00	0.04	0.00	*0.91*	40
Unknown	*0.13*	*0.12*	*0.37*	*0.04*	*0.02*	*0.22*	*0.11*	3424
ADMIXTURE^1^	POP1	POP2	POP3	POP4	POP5	POP6	POP7	n_p_
NE	0.05	0.09	*0.42*	0.08	0.06	0.14	0.16	601
SE	0.05	0.08	0.06	*0.41*	0.21	0.11	0.08	100
GB	0.06	0.09	0.16	0.07	0.07	*0.48*	0.08	124
Russia	0.05	0.07	0.16	0.06	0.08	0.04	*0.54*	13
Arab	0.05	0.38	0.03	0.10	*0.40*	0.02	0.02	62
Basque	0.05	0.05	0.07	*0.42*	0.03	0.28	0.09	12
Jew	*0.62*	0.06	0.07	0.05	0.10	0.05	0.06	40
Unknown	*0.12*	*0.08*	*0.16*	*0.14*	*0.12*	*0.26*	*0.12*	3424
ADMIXTURE^2^	POP1	POP2	POP3	POP4	POP5	POP6	POP7	n_p_
NE	0.00	0.00	0.00	0.00	0.00	1.00*	0.00	601
SE	1.00*	0.00	0.00	0.00	0.00	0.00	0.00	100
GB	0.00	0.00	0.00	0.00	1.00*	0.00	0.00	124
Russia	0.00	0.00	0.00	0.00	0.00	0.00	1.00*	13
Arab	0.00	0.00	0.00	1.00*	0.00	0.00	0.00	62
Basque	0.00	0.00	1.00*	0.00	0.00	0.00	0.00	12
Jew	0.00	1.00*	0.00	0.00	0.00	0.00	0.00	40
Unknown	*0.14*	*0.13*	*0.08*	*0.07*	*0.31*	*0.18*	*0.09*	3424

Note that superscripts a-g indicate the proportions inferred from each population centroid. Superscript^1^ and superscript^2^ are computed without and with population identities, respectively. The number in bracket presents the number of admixtures in AIPS. The italicized number presents the highest correct classification rates for each population. *The ancestry inference with asterisk was obtained by supervised learning mode in ADMIXTURE, assigning 100% ancestry membership without further computation

where ***n***_***p***_ is a number of individuals in each population group, ***p*** a number of population group, and ***Avg*** % ***Correct***_***i***_ the correct population proportion for each individual. The average of Avg%Correct using AIPS is 0.81 among 7 population groups. As presented in Table 2, AIPS has correct classification rates between 0.68–0.90 for inferring the correct ancestry memberships whereas STRUCTURE without population information identifies correct classification between 0.21–0.64 and ADMIXTURE identifies correct classification between 0.37–0.92 among 7 European and closely related subpopulation clusters. It is not easy to identify and match true ancestry clusters in results from STRUCTURE when there are no distinct patterns between similarity and dissimilarity. Although STRUCTURE with prior population assignment identities has higher correct classification rates in the range of 0.46–1.00 than STRUCTURE without prior assignment in 0.21–0.64, STRUCTURE with prior assignment assigns only three major clusters; each cluster consists of 4, 2, and 1 subpopulations, respectively; the first cluster includes Northern European (NE, 0.93), Great Britain (GB, 1.00), Russian (1.00), and Basque (0.64); the second one includes Southern European (SE, 0.46) and Jew (1.00); the last one includes Arab (0.89). Europeans are commonly considered as a largely homogeneous population by STRUCTURE. AIPS can detect the distinction among NE, GB, Russia, and Basque while STRUCTURE is unable to distinguish among them. ADMIXTURE using supervised learning mode requires an additional file, specifying the ancestries of the reference (known) individuals. ADMIXTURE assigns 100% ancestry membership to all reference samples without further computation. We performed further comparison including 3424 Europeans with unknown subpopulation information between AIPS and ADMIXTURE. AIPS enables one to identify the ethnic heterogeneity whereas ADMIXTURE cannot recognize genetic dissimilarity between SE and Basque. According to Fig. 2 (b), there are a very small number of unknown samples of apparent Arab descent (in pink); AIPS assigned 2% out of 3424 samples into Arab subpopulation, while ADMIXTURE with and without reference information inferred 12 and 7% as Arab, respectively. In addition, we compared the average of Avg%Correct with AIPS and ADMIXTURE among 22 European subpopulations. AIPS assuming 3 admixtures has correct classification rates within 0.18–0.89 for inferring the correct ancestry memberships whereas ADMIXTURE without reference information identifies within 0.09–0.56 among 22 European subpopulations (Additional file 2: Table S4). AIPS identifies the genetic heterogeneity among 20 populations except CEU (0.34) and Italian (0.18) while ADMIXTURE clusters 22 subpopulations into 10 subpopulations, presenting no distinction in small genetic differences. Therefore, the assignment to subpopulations by AIPS outperforms the commonly used approaches, STRUCTURE and ADMIXTURE with or without prior (reference) subpopulation information.

## Discussion

Population stratification in genome-wide association studies can result in many false-positive discoveries and mask the true associations [21]. Sometimes, genetic ancestry may not be available to the researchers and even though available, it may not be accurate for the underlying population genetic structure from self-reported questionnaire. It is important to confirm if self-reported ethnicity is correct and to infer the correct genetic ancestry of uncategorized individuals in many scientific studies.

The most common tool for accounting for the confounding effects of population stratification is principal component analysis (PCA). When the sample size is small, applying PCA is simple. However, because genomic high-throughput technologies are advancing, we now have larger data sets that are more difficult to analyze, especially related to inferring genetic ancestry. The widely used tool for detecting and adjusting population stratification is EIGENSOFT including two features; EIGENSTRAT and smartpca. The downside to EIGENSOFT is unable to provide correct ancestral origins while AIPS enables one to predict ancestry memberships with PCA scores as an input. The scores from PCA explain the similar patterns between samples and the eigenvectors called SNP weights (loadings) similarity between variables. Thus, the PCA scores can be used to adjust for population structures and identify ethnic origins in GWAS.

There are two types of ancestry inference approaches; distance-based and model-based approaches. STRUCTURE and fastSTRUCTURE are the typical example of model-based approach. Model-based approach adapts parametric model; Bayesian or maximum likelihood method. For example, STRUCTURE uses the characteristic set of allele frequencies, Hardy-Weinberg equilibrium and complete linkage equilibrium between loci within populations to compute the ancestry inference in MCMC algorithm. Alternative approaches based on distance (similarity) matrix are GSM, Spectral-GEM, and FastPop. GSM and Spectral-GEM calculated the similarity matrix based on IBS measures and distance between two subjects that require computational intensity when the sample size is very large. FastPop results in complex computation and has not been established when inferring genetic ancestry among more than 4 population substructures. AIPS is a distance-based approach and very straightforward to infer ancestry origins. It combines two widely used statistical methods that are principal component analysis and spatial analysis. First, we compute scores of individuals and the centroid of each population in PCA and manipulate spatial information to extract distance relationship information in spatial analysis. The simplest spatial interpolation method, the inverse distance weighted interpolation is applied. This reveals the closeness between each centroid and score of individual. The calculation is very simple and straightforward and consequently the computational speed is faster. AIPS is a similar method compared to other existing population inference tools for estimating global ancestry membership like fastSTRUCTURE, because the eigenvectors from the covariance matrix are maximum likelihood estimators [24, 29]. Nevertheless, AIPS is comparably faster and achieves more accurate validation. For 952 samples using 25,732 ancestry informative markers, AIPS finished the ancestry inferences in less than 5 min to get principal components and less than 1 min to infer ancestry memberships compared with 19–23 h required by STRUCTURE, 3–4 h by fastSTRUCTURE for both 7 and 22 subpopulations, and about 20 min for 7 subpopulations and 6.5 h for 22 subpopulations by ADMIXTURE. For 4376 samples, AIPS took about 1 h to compute principal components and less than 2 min to make ancestry inferences for both 7 and 22 subpopulations while ADMIXTURE required about 28 min with reference information and about 5.5 h without reference information for 7 populations and 136.35 h without reference information for 22 populations. Furthermore, the heuristic ranks to closeness among each centroid of subpopulation provide a reasonably geogenetic relationship map to assign the given large subpopulations into the smaller clusters.

In this paper, we provide a distinct and reasonable population inference framework that achieves better accuracy comparable to STRUCTURE and fastSTRUCTURE with faster computational speed. While STRUCTURE and fastSTRUCTURE take quite long time to infer individual’s ancestry membership, AIPS takes about an hour to calculate the distance metrics of substructures for ancestry inference among 4376 individuals on 25,732 AIMs. In addition, AIPS allows one to choose the number of admixtures and top ranked eigenvalues that reflect the proportion of total variance explained by the eigenvectors. Plotting eigenvalues indicates how many top ranked eigenvalues should be included in the analysis.

If consortiums generate the large number of samples and would like to perform consistent approach, computing and sharing SNP weights (loading) consisting of the similarity in the markers (SNPs) on the specific set of AIMs are recommended. SNP weights on specific AIMs enable to predict the new variance components (scores) in new data that improves the computational efficiency and provide the consistent approach to perform multiple independent analyses in the large consortia. We recommend that the number of samples should be greater than one of markers due to shrinkage issue. In the case of analyzing genotyped data generated from same platform, AIPS can predict scores of new samples projected from SNP weights, which are eigenvectors, on the same pre-defined AIMs. This is an efficient computational framework to account for the confounding effects of population stratification and infer individual genetic ancestry in large consortiums.

For illustration, we selected population substructures in Europe. Europeans including European-Americans are considered as a single ethnic group such as “White” or “Caucasian” in many surveys [30]. In reality, Europeans have historically diverse ancestry and their genetic structure is strongly correlated with their geographical location [31]^.^ We demonstrated intra-European analysis involving 4376 individuals on 25,732 intra-European AIMs. Among them, 952 samples represented 22 ancestry-known subpopulations. We presented the comparisons among AIPS, STRUCTURE, and fastSTRUCTURE in graphical displays. In addition, we reduced the number of subpopulations to check the accuracy of ancestry classification. The reduced 7 clusters from 22 subpopulations within Europe are clearly distinct as suggested by Hotelling’s T^2^ test. We evaluated them with average of correctly inferred proportions. AIPS improves the level of accuracy for inferring ancestry memberships.

Better implementation of AIPS benefits from the choice of publicly available subpopulations. A pairwise distance matrix obtained between each subject and centroids of the known population substructures provides more accurate and clearer interpretation of the underlying substructures.

## Conclusions

Genome-wide association studies in the high-density single-nucleotide polymorphism genotyping data have identified thousands of common variants associated to complex disease risks and traits. Because the frequency difference in genetic population structure between cases and controls due to systematic ancestry difference can lead to false-positive results, an accurate inference of genetic ethnic membership is extremely important in many biomedical research areas. Although a few applications for detecting stratification and estimating genetic ancestry in population genetics have been developed, applying them to large genetic studies is challenging in computational time and cost. Analyzing large genotyped samples, which are becoming increasingly available, with self-reported or unknown ancestry labels, AIPS can improve accuracy in estimating ancestry memberships as well as computation efficiency. The R-package AIPS will be available for downloading at https://morgan.dart-mouth.edu/~f000q4v/html/aips.html.

## Additional files

Additional file 1: Supplementary Methods. Mathematical definition of principal component analysis. (DOCX 20 kb)

Additional file 2: R-Supplementary Materials. The attached file includes 4 supplementary figures and 4 supplementary tables. **Figure S1.** C_N.Euro_ and C_Rus_ present centroid of known ancestry samples from Northern Europeans and Russians, respectively. (a) With the first three scores from PCA, individual **A** seems to be closer to Russian group on the proportion of total variance explained by eigenvalues. (b) In the two dimensional plot with the top two principal component scores, individual **A** seems to be closer to N. European. **Figure S2.** Comparison of eigenvalues and top 3 principal components from AIPS and EIGENSTRAT. The options that were set in EIGENSTRAT were numoutlieriter = 0;outliermode = 2(no outlier removal) and in AIPS the option was method = eigen. **Figure S3.** Graphical Comparison of Population Structure using AIPS among 22 European subpopulations. Only 952 known ancestry individuals were used in 22 subpopulations within Europe. The scores from PCA were first calculated then Inverse-Distance Weighted Interpolation without and with eigenvalue weight were applied to infer the ancestry membership. The number of admixture indicated the definition of admixture in AIPS. **Figure S4.** Graphical Comparison of Population Structure using STRUCTURE and fastSTRUCTURE among 22 European subpopulations. The inferences of ancestry membership for 952 individuals were calculated by STRUCTURE and fastSTRUCTURE. (a) The prior population information was not given to compute inference of population membership using STRUCTURE. (b) To infer the population membership within 22 Europe countries, the prior population information was assigned in STRUCTURE. (c) fastSTRUCTURE was applied to infer 22 European subpopulations with simple model. (d) fastSTRUCTURE was used with logistic prior model. **Table S1.** Distance-based clustering among 952 known and 3426 unknown ancestry Europeans on 25,732 AIMs. **Table S2.** Rank-based on Closeness among 22 European subpopulations. **Table S3.** Distance between two centroids among 22 European subpopulations. **Table S4.** The Average Percent of Correctly Inferred Proportions from AIPS and ADMIXTURE without Population Information. (DOCX 922 kb)

## Abbreviations

AIMs: Ancestry Informative Markers
AIPS: Ancestry Inference using Principal component analysis and Spatial analysis
ED: Exponential-Distance
GB: Great Britain
GEM: Genetic Matching
GSM: Genetic Similarity Score Matching
GWAS: Genome-Wide Association Studies
HGDP: Human Genome Diversity Project
IBS: Identity-By-State
IDW: Inverse Distance Weighted
NE: Northern European
PCA: Principal Component Analysis
PD: Power-Distance
SE: Southern European

## References

[1] Amos CI, Wang LE, Lee J (2011). Genome-wide association study identifies novel loci predisposing to cutaneous melanoma. Hum Mol Gen.

[2] Devlin B, Roeder K (1999). Genomic control for association studies. Biometrics.

[3] Price AL, Zaitlen NA, Reich D, Patterson N (2010). New approaches to population stratification in genome-wide association studies. Nature Rev Genet.

[4] Tian C, Gregersen PK, Seldin MF (2008). Accounting for ancestry: population substructure and genome-wide association studies. Hum Mol Gen.

[5] Wacholder S, Rothman N, Caporaso N (2002). Counterpoint: bias from population stratification is not a major threat to the validity of conclusions from epidemiological studies of common polymorphisms and cancer. Cancer Epidemiol Biomarkers Prevent.

[6] Amirisetty S, Hershey GKK, Baye TM (2012). AncestrySNPminer: a bioinformatics tool to retrieve and develop ancestry informative SNP panels. Genomics.

[7] Baye TM, Tiwari HK, Allison DB, Go RC. Database mining for selection of SNP markers useful in admixture mapping. BMC BioData Mining. 2009; doi:10.1186/1756-0381-2-1. 10.1186/1756-0381-2-1 PMC2649128 19216798

[8] Kodaman N, Aldrich MC, Smith JR (2013). A small number of candidate gene SNPs reveal continental ancestry in African Americans. Ann Hum Genet.

[9] Kosoy R, Nassir R, Tian C (2009). Ancestry informative marker sets for determining continental origin and admixture proportions in common populations in America. Hum Mutat.

[10] Ma J, Amos CI. Principal components analysis of population admixture. PLoS One. 2012;7(7):e40115. doi:10.1371/journal.pone.0040115. 10.1371/journal.pone.0040115 PMC3392282 22808102

[11] Pardo-Seco J, Martinon-Torres F, Salas A (2014). Evaluating the accuracy of AIM panels at quantifying genome ancestry. BMC Genomics.

[12] Tian C, Plenge RM, Ransom M, et al. Analysis and application of European genetic substructure using 300 K SNP information. PLoS Genet. 2008; doi: 10.1371/journal.pgen.0040004. 10.1371/journal.pgen.0040004 PMC2211544 18208329

[13] Tian C, Kosoy R, Nassir R (2009). European population genetic substructure: further definition of ancestry informative markers for distinguishing among diverse European ethnic groups. Mol Med.

[14] Menozzi P, Piazza A, Cavalli-Sforza L (1978). Synthetic maps of human gene frequencies in Europeans. Science.

[15] Patterson N, Price AL, Reich D. Population Structure and Eigenanalysis. PLoS Genetics. 2006; doi: 10.1371/journal.pgen.0020190. 10.1371/journal.pgen.0020190 PMC1713260 17194218

[16] Price AL, Patterson NJ, Plenge RM (2006). Principal components analysis corrects for stratification in genome-wide association studies. Nature Genet.

[17] Pritchard JK, Stephens M, Donnelly P (2000). Inference of population structure using multilocus genotype data. Genetics.

[18] Raj A, Stephens M, Pritchard JK (2014). fastSTRUCTURE: Variational inference of Populatioin structure in large SNP data sets. Genetics.

[19] Alexander DH, Novembre J, Lange K (2009). Fast model-based estimation of ancestry in unrelated individuals. Genome Res.

[20] Alexander DH, Lange K (2011). Enhancements to the ADMIXTURE algorithm for individual ancestry estimation. BMC Bioinformatics.

[21] Guan W, Liang L, Boehnke M, Abecasis GR (2009). Genotype-based matching to correct for population stratification in large-scale case-control genetic association studies. Genet Epidemiol.

[22] Lee AB, Luca D, Klei L (2010). Discovering genetic ancestry using spectral graph theory. Genet Epidemiol.

[23] Li Y, Byun J, Cai G (2016). FastPop: a rapid principal component derived method to infer intercontinental ancestry using genetic data. BMC Bioinformatics.

[24] Lee S, Zou F, Wright FA (2010). Convergence and prediction of principal component scores in high-dimensional settings. Ann Stat.

[25] Amos CI, Dennis J, Wang Z, Byun J (2017). The OncoArray consortium: a network for understanding the genetic architecture of common cancers. Cancer Epidemiol Biomark Prev.

[26] Sudlow C, Gallacher J, Allen N, Beral V (2015). UK biobank: an open access resource for identifying the causes of a wide range of complex diseases of middle and old age. PLoS Med.

[27] Novembre J, Johnson T, Bryc K (2008). Genes mirror geography within Europe. Nature.

[28] Porras-Hurtado L, Ruiz Y, Santos C, Phillips C (2013). An overview of STRUCTURE: applications, parameter settings, and supporting software. Front Genet.

[29] Girshick M (1936). Principal components. JASA.

[30] Price AL, Butler J, Patterson N (2008). Discerning the ancestry of European Americans in genetic association studies. PLoS Genet.

[31] Nelis M, Esko T, Magi R (2009). Genetic structure of Europeans: a view from the north-east. PLoS One.

