# Supplementary material for: Ancestry inference using principal component analysis and spatial analysis: a distance-based analysis to account for population substructure
Source: BMC Genomics. 2017 Oct 16;18:789. doi: 10.1186/s12864-017-4166-8 (PMC5644186; doi:10.1186/s12864-017-4166-8)
Supplement: Supplementary file 1 — Supplementary Methods. Mathematical definition of principal component analysis. (DOCX 20 kb) [file 12864_2017_4166_MOESM1_ESM.docx]

**Supplementary Methods**

Let $\boldsymbol{X}_{\boldsymbol{n}\times\boldsymbol{p}}=\left( \begin{matrix} \boldsymbol{x}_{\mathbf{11}} & \cdots& \boldsymbol{x}_{\mathbf{1}\boldsymbol{p}} \\ \vdots& \ddots& \vdots\\ \boldsymbol{x}_{\boldsymbol{n}\mathbf{1}} & \cdots& \boldsymbol{x}_{\boldsymbol{np}} \end{matrix} \right)$ be a sample matrix, where ***n*** and ***p*** are the number of individuals and SNPs, respectively. For the mean-centered sample matrix, compute the mean on each SNP, ${\bar{\boldsymbol{x}}}_{.\boldsymbol{j}}=\frac{\sum_{\boldsymbol{i}=\mathbf{1}}^{\boldsymbol{n}} \boldsymbol{x}_{\boldsymbol{ij}}}{\boldsymbol{n}} ,\boldsymbol{i}=\mathbf{1},\cdots,\boldsymbol{n}\mathrm{and}\boldsymbol{j}=\mathbf{1},\cdots,\boldsymbol{p}.$

Then, the mean-centered data matrix is written by

$\boldsymbol{Y}_{\boldsymbol{n}\times\boldsymbol{p}} \equiv\left[ \begin{matrix} \boldsymbol{x}_{\mathbf{11}}-{\bar{\boldsymbol{x}}}_{.\mathbf{1}} & \cdots& \boldsymbol{x}_{\mathbf{1}\boldsymbol{p}}-{\bar{\boldsymbol{x}}}_{.\boldsymbol{p}} \\ \vdots& \ddots& \vdots\\ \boldsymbol{x}_{\boldsymbol{n}\mathbf{1}}-{\bar{\boldsymbol{x}}}_{.\mathbf{1}} & \cdots& \boldsymbol{x}_{\boldsymbol{np}}-{\bar{\boldsymbol{x}}}_{.\boldsymbol{p}} \end{matrix} \right]$ .

For $\boldsymbol{n}>\boldsymbol{p}$ (larger number of individuals than SNPs), construct the covariance matrix as

$$\boldsymbol{C}_{\boldsymbol{p}\times\boldsymbol{p}}= \frac{\mathbf{1}}{\boldsymbol{n}-\mathbf{1}} \cdot\boldsymbol{Y}^{\boldsymbol{T}}\boldsymbol{Y} .$$

Since the covariance matrix ***C*** is symmetric and positive definite, the eigenvalues of covariance matrix ***C*** are real and positive semi-definite.

For calculating the eigenvectors and eigenvalues from the covariance matrix ***C***, the spectral decomposition or the singular value decomposition of a matrix ***C*** can be applied. The eigenvalues $\boldsymbol{\lambda}_{\boldsymbol{i}}$ and the eigenvectors $\boldsymbol{v}_{\boldsymbol{i}}$ of ***C*** satisfy that $\boldsymbol{C}{\underline{\boldsymbol{v}}}_{\boldsymbol{i}}=\boldsymbol{\lambda}_{\boldsymbol{i}}{\underline{\boldsymbol{v}}}_{\boldsymbol{i}}.$

Rewrite the last equation in terms of matrix form as $\boldsymbol{CV}=\boldsymbol{V\Lambda} ,$

where $\boldsymbol{\Lambda}_{\boldsymbol{p}\times\boldsymbol{p}}=\left[ \begin{matrix} \begin{matrix} \begin{matrix} \boldsymbol{\lambda}_{\mathbf{1}} \\ \mathbf{0} \end{matrix} & \begin{matrix} \mathbf{0} \\ \boldsymbol{\lambda}_{\mathbf{2}} \end{matrix} & \begin{matrix} \mathbf{0} \\ \mathbf{0} \end{matrix} \end{matrix} & \cdots& \begin{matrix} \mathbf{0} \\ \mathbf{0} \end{matrix} \\ \vdots& \ddots& \vdots\\ \begin{matrix} \mathbf{0} & \mathbf{0} & \mathbf{0} \end{matrix} & \cdots& \boldsymbol{\lambda}_{\boldsymbol{p}} \end{matrix} \right]$ is the diagonal matrix with the eigenvalues $\boldsymbol{\lambda}_{\boldsymbol{i}}$ in the diagonal.

In PCA, the $\boldsymbol{\lambda}_{\boldsymbol{i}}$ in $\boldsymbol{\Lambda}$ are assigned with the descent order (i.e. $\boldsymbol{\lambda}_{\mathbf{1}}\geq\boldsymbol{\lambda}_{\mathbf{2}}\geq\cdots\geq\boldsymbol{\lambda}_{\boldsymbol{p}}$) and the matrix $\boldsymbol{V}_{\boldsymbol{p}\times\boldsymbol{p}}=\left( \begin{matrix} \begin{matrix} {\underline{\boldsymbol{v}}}_{\mathbf{1}} & {\underline{\boldsymbol{v}}}_{\mathbf{2}} \end{matrix} & \begin{matrix} \cdots& {\underline{\boldsymbol{v}}}_{\boldsymbol{p}} \end{matrix} \end{matrix} \right)$consists of eigenvectors corresponding to $\boldsymbol{\lambda}_{\boldsymbol{i}}$ .

Thus, the spectral decomposition of the covariance matrix ***C*** is

$\boldsymbol{C}=\boldsymbol{V\Lambda}\boldsymbol{V}^{-\mathbf{1}}=\boldsymbol{V\Lambda}\boldsymbol{V}^{\boldsymbol{T}}$

and because ***C*** is a correlation matrix, ***C*** can be only positive definite when selecting independent markers.

Since ***C*** is symmetric and positive definite, $\boldsymbol{V}^{\boldsymbol{T}}=\boldsymbol{V}^{-\mathbf{1}}.$

Finally, to reduce the dimension in PCA, we can pick an arbitrary number $\boldsymbol{k} \leq\boldsymbol{p}$ for the first $\boldsymbol{k}$ principal components with projection matrix comprised of eigenvectors, ${\underline{\boldsymbol{v}}}_{\mathbf{1}}, \cdots, {\underline{\boldsymbol{v}}}_{\boldsymbol{k}}$. When analyzed in P-mode (eigen), the eigenvectors can be used to score SNPs and may be called SNP weights,

$$\boldsymbol{E}_{\boldsymbol{p}\times\boldsymbol{k}}= \left( \begin{matrix} \begin{matrix} {\underline{\boldsymbol{v}}}_{\mathbf{1}} & {\underline{\boldsymbol{v}}}_{\mathbf{2}} \end{matrix} & \begin{matrix} \cdots& {\underline{\mathbf{v}}}_{\boldsymbol{k}} \end{matrix} \end{matrix} \right) .$$

Thus, we can obtain the low dimensional projection, score matrix, $\boldsymbol{Z}_{\boldsymbol{n}\times\boldsymbol{k}}=\boldsymbol{Y}_{\boldsymbol{n}\times\boldsymbol{p}}\times\boldsymbol{E}_{\boldsymbol{p}\times\boldsymbol{k}}$ .

For $\boldsymbol{n}<\boldsymbol{p}$ (larger number of SNPs than individuals) where $\boldsymbol{p}\times\boldsymbol{p}$ covariance matrix does not have full rank it is required to construct an alternative covariance matrix with the lower dimension of $\boldsymbol{n}\times\boldsymbol{n}$; $\boldsymbol{D}_{\boldsymbol{n}\times\boldsymbol{n}}= \frac{\mathbf{1}}{\boldsymbol{n}-\mathbf{1}} \cdot\boldsymbol{Y}\boldsymbol{Y}^{\boldsymbol{T}}$. From the covariance matrix ***D***, we get the following equation; $\boldsymbol{Y}\boldsymbol{Y}^{\boldsymbol{T}}{{\underline{\boldsymbol{v}}}_{\boldsymbol{i}}}^{*}={\boldsymbol{\lambda}_{\boldsymbol{i}}}^{*}{{\underline{\boldsymbol{v}}}_{\boldsymbol{i}}}^{*}$,

then $\boldsymbol{Y}^{\boldsymbol{T}}\boldsymbol{Y}\boldsymbol{Y}^{\boldsymbol{T}}{{\underline{\boldsymbol{v}}}_{\boldsymbol{i}}}^{*}={\boldsymbol{\lambda}_{\boldsymbol{i}}}^{*}\boldsymbol{Y}^{\boldsymbol{T}}{{\underline{\boldsymbol{v}}}_{\boldsymbol{i}}}^{*}$ is proportional to $\boldsymbol{C}\boldsymbol{Y}^{\boldsymbol{T}}{{\underline{\boldsymbol{v}}}_{\boldsymbol{i}}}^{*}={\boldsymbol{\lambda}_{\boldsymbol{i}}}^{*}\boldsymbol{Y}^{\boldsymbol{T}}{{\underline{\boldsymbol{v}}}_{\boldsymbol{i}}}^{*}$ .

Similarly, $\boldsymbol{C}{\underline{\boldsymbol{v}}}_{\boldsymbol{i}}= \frac{\mathbf{1}}{\boldsymbol{N}-\mathbf{1}} \cdot\boldsymbol{Y}^{\boldsymbol{T}}\boldsymbol{Y}{\underline{\boldsymbol{v}}}_{\boldsymbol{i}} =\boldsymbol{\lambda}_{\boldsymbol{i}}{\underline{\boldsymbol{v}}}_{\boldsymbol{i}}$ yields $\boldsymbol{Y}^{\boldsymbol{T}}\boldsymbol{Y}{\underline{\boldsymbol{v}}}_{\boldsymbol{i}} =\boldsymbol{\lambda}_{\boldsymbol{i}}{\underline{\boldsymbol{v}}}_{\boldsymbol{i}}$.

Then we can have $\boldsymbol{Y}^{\boldsymbol{T}}\boldsymbol{Y}{\underline{\boldsymbol{v}}}_{\boldsymbol{i}} =\boldsymbol{\lambda}_{\boldsymbol{i}}\boldsymbol{Y}{\underline{\boldsymbol{v}}}_{\boldsymbol{i}}$ , which is equivalent to$\boldsymbol{DY}{\underline{\boldsymbol{v}}}_{\boldsymbol{i}}=\boldsymbol{\lambda}_{\boldsymbol{i}}\boldsymbol{Y}{\underline{\boldsymbol{v}}}_{\boldsymbol{i}}$ . From the last equation, $\boldsymbol{\lambda}_{\boldsymbol{i}}$ is an eigenvalue with corresponding eigenvector $\boldsymbol{Y}{\underline{\boldsymbol{v}}}_{\boldsymbol{i}}$ from covariance matrix ***D***.

Both $\boldsymbol{Y}\boldsymbol{Y}^{\boldsymbol{T}}$and $\boldsymbol{Y}^{\boldsymbol{T}}\boldsymbol{Y}$are symmetric and positive definite matrix and $\boldsymbol{rank}\left( \boldsymbol{Y}\boldsymbol{Y}^{\boldsymbol{T}} \right)=\boldsymbol{rank}\left( \boldsymbol{Y}^{\boldsymbol{T}}\boldsymbol{Y} \right)=\boldsymbol{rank}\left( \boldsymbol{Y} \right)$. Thus, $\boldsymbol{Y}^{\boldsymbol{T}}\boldsymbol{Y}$ and $\boldsymbol{Y}\boldsymbol{Y}^{\boldsymbol{T}}$ have same set of eigenvalues.

The same number of eigenvectors corresponding to nonzero eigenvalues can be obtained by $\boldsymbol{V}_{\boldsymbol{P}\times\boldsymbol{N}}=\boldsymbol{Y}_{\boldsymbol{p}\times\boldsymbol{n}}^{\boldsymbol{T}}\times\boldsymbol{V}_{\boldsymbol{n}\times\boldsymbol{n}}^{*}$ , where $\boldsymbol{V}_{\boldsymbol{n}\times\boldsymbol{n}}^{*}$ is the eigenvectors from covariance matrix ***D***. After applying these computational steps, we can compute the score matrix ***S*** in terms of the largest k principal components, $\boldsymbol{S}_{\boldsymbol{n}\times\boldsymbol{k}}=\boldsymbol{Y}_{\boldsymbol{n}\times\boldsymbol{p}}\times\boldsymbol{E}_{\boldsymbol{p}\times\boldsymbol{k}}$ , where $\boldsymbol{E}_{\boldsymbol{p}\times\boldsymbol{k}}$is the matrix with the first k eigenvectors.

Once we select the first k eigenvectors, named as SNP weights, which we would like to keep among the principal components computed from the discovery data, we can predict new scores in the new data using pre-computed SNP weights.

Let $\boldsymbol{U}_{\boldsymbol{m}\times\boldsymbol{p}}=\left( \begin{matrix} \boldsymbol{u}_{\mathbf{11}} & \cdots& \boldsymbol{u}_{\mathbf{1}\boldsymbol{p}} \\ \vdots& \ddots& \vdots\\ \boldsymbol{u}_{\boldsymbol{m}\mathbf{1}} & \cdots& \boldsymbol{u}_{\boldsymbol{m}p} \end{matrix} \right)$ be a new data with ***m*** samples and the same ***p*** SNPs as in the original analysis.

Then, generate the mean-centered matrix, $\boldsymbol{W}_{\boldsymbol{m}\times\boldsymbol{p}} \equiv\left[ \begin{matrix} \boldsymbol{u}_{\mathbf{11}}-{\bar{\boldsymbol{u}}}_{.\mathbf{1}} & \cdots& \boldsymbol{u}_{\mathbf{1}\boldsymbol{p}}-{\bar{\boldsymbol{u}}}_{.\boldsymbol{p}} \\ \vdots& \ddots& \vdots\\ \boldsymbol{u}_{\boldsymbol{m}\mathbf{1}}-{\bar{\boldsymbol{u}}}_{.\mathbf{1}} & \cdots& \boldsymbol{u}_{\boldsymbol{mp}}-{\bar{\boldsymbol{u}}}_{.\boldsymbol{p}} \end{matrix} \right]$ .

Using the SNP weights$\boldsymbol{E}_{\boldsymbol{p}\times\boldsymbol{k}}$ from the original analysis, compute the new score matrix $\boldsymbol{S}^{\boldsymbol{*}}$,

${\boldsymbol{S}^{\boldsymbol{*}}}_{\boldsymbol{m}\boldsymbol{\times}\boldsymbol{k}}\boldsymbol{=}\boldsymbol{W}_{\boldsymbol{m}\boldsymbol{\times}\boldsymbol{p}}\boldsymbol{\times}\boldsymbol{E}_{\boldsymbol{p}\boldsymbol{\times}\boldsymbol{k}}$.
