# Supplementary material for: Ancestry inference using principal component analysis and spatial analysis: a distance-based analysis to account for population substructure
Source: BMC Genomics. 2017 Oct 16;18:789. doi: 10.1186/s12864-017-4166-8 (PMC5644186; doi:10.1186/s12864-017-4166-8)
Supplement: Supplementary file 2 — R-Supplementary Materials. The attached file includes 4 supplementary figures and 4 supplementary tables. Figure S1. CN.Euro and CRus present centroid of known ancestry samples from Northern Europeans and Russians, respectively. (a) With the first three scores from PCA, individual A seems to be closer to Russian group on the proportion of total variance explained by eigenvalues. (b) In the two dimensional plot with the top two principal component scores, individual A seems to be closer to N. European. Figure S2. Comparison of eigenvalues and top 3 principal components from AIPS and EIGENSTRAT. The options that were set in EIGENSTRAT were numoutlieriter = 0;outliermode = 2(no outlier removal) and in AIPS the option was method = eigen. Figure S3. Graphical Comparison of Population Structure using AIPS among 22 European subpopulations. Only 952 known ancestry individuals were used in 22 subpopulations within Europe. The scores from PCA were first calculated then Inverse-Distance Weighted Interpolation without and with eigenvalue weight were applied to infer the ancestry membership. The number of admixture indicated the definition of admixture in AIPS. Figure S4. Graphical Comparison of Population Structure using STRUCTURE and fastSTRUCTURE among 22 European subpopulations. The inferences of ancestry membership for 952 individuals were calculated by STRUCTURE and fastSTRUCTURE. (a) The prior population information was not given to compute inference of population membership using STRUCTURE. (b) To infer the population membership within 22 Europe countries, the prior population information was assigned in STRUCTURE. (c) fastSTRUCTURE was applied to infer 22 European subpopulations with simple model. (d) fastSTRUCTURE was used with logistic prior model. Table S1. Distance-based clustering among 952 known and 3426 unknown ancestry Europeans on 25,732 AIMs. Table S2. Rank-based on Closeness among 22 European subpopulations. Table S3. Distance between two centroids among 22 E [file 12864_2017_4166_MOESM2_ESM.docx]

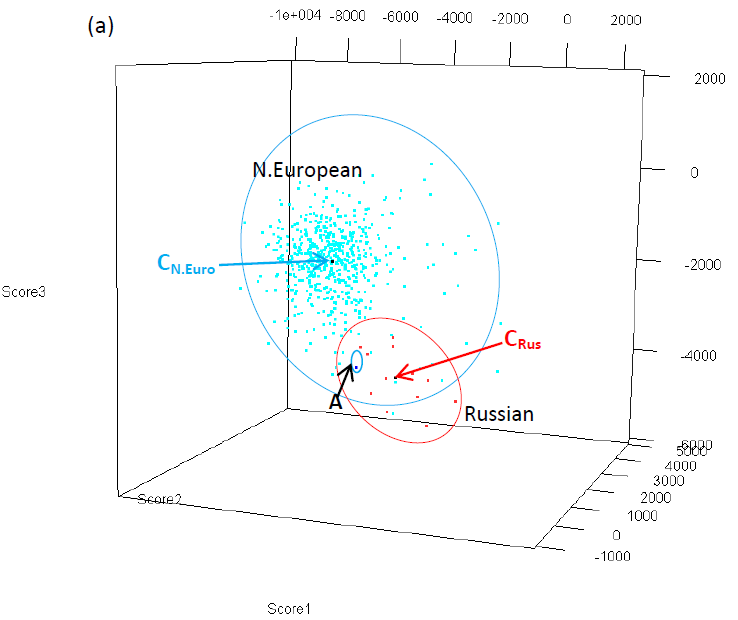

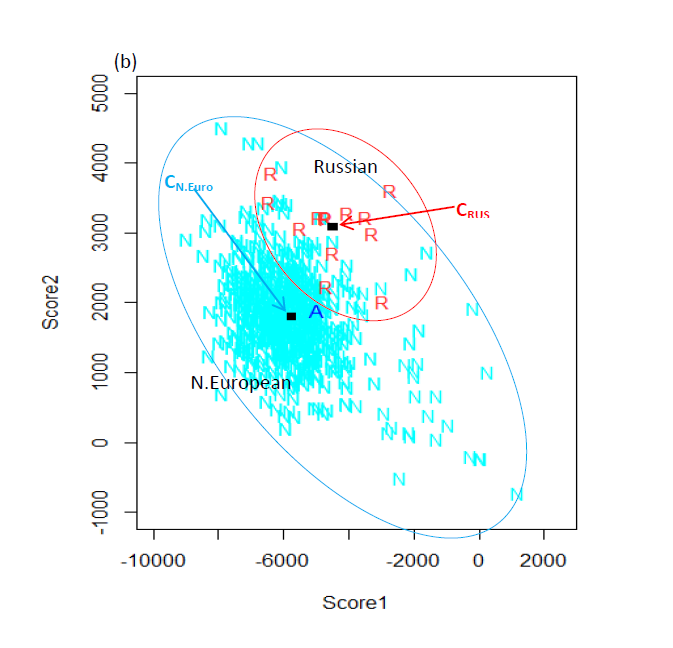


**Supplementary Figure 1** C_N.Euro_ and C_Rus_ present centroid of known ancestry samples from Northern Europeans and Russians, respectively. (a) With the first three scores from PCA, individual **A** seems to be closer to Russian group on the proportion of total variance explained by eigenvalues. (b) In the two dimensional plot with the top two principal component scores, individual **A** seems to be closer to N. European.


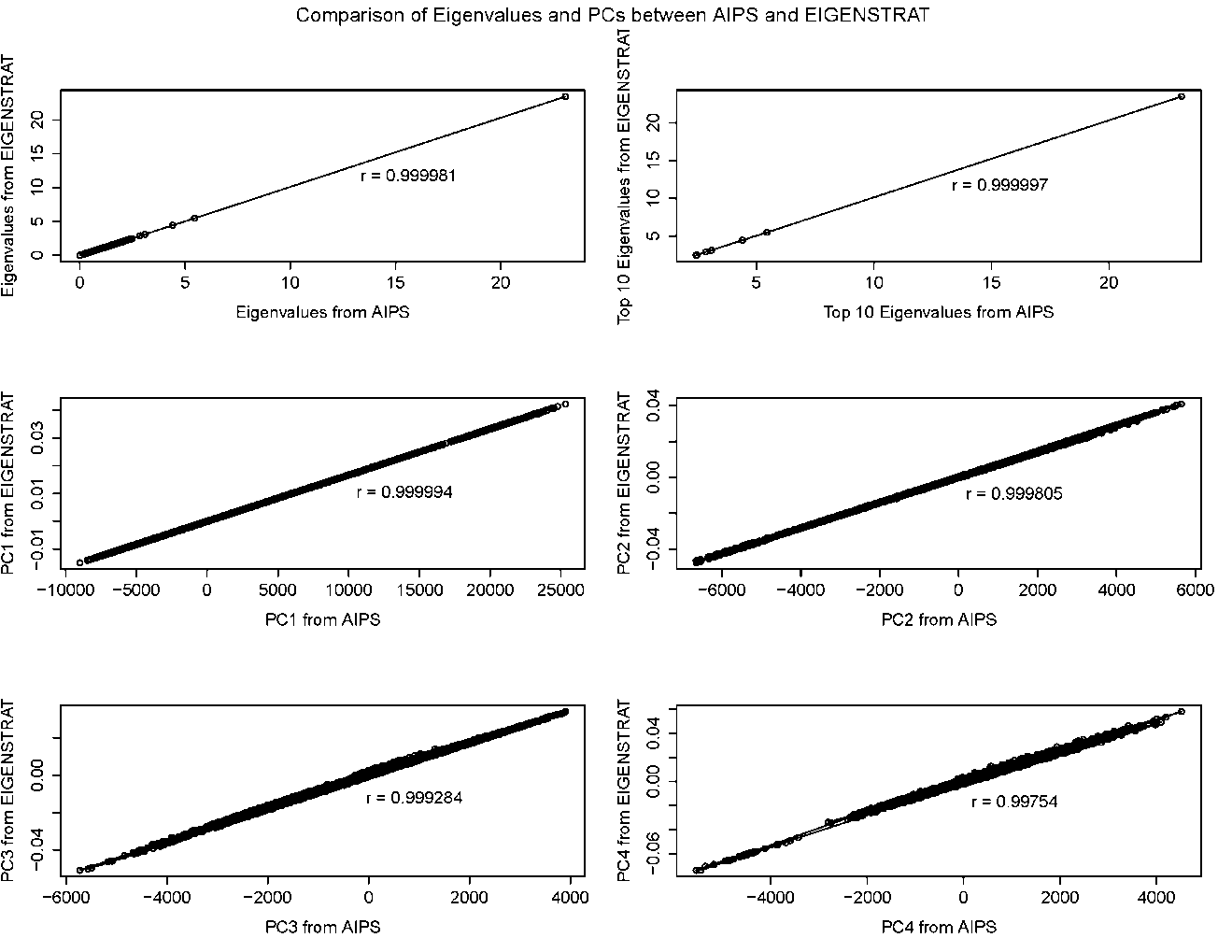


**Supplementary Figure 2** Comparison of eigenvalues and top 3 principal components from AIPS and EIGENSTRAT. The options that were set in EIGENSTRAT were numoutlieriter=0;outliermode=2(no outlier removal) and in AIPS the option was method=eigen.

**Supplementary Figure 3** Graphical Comparison of Population Structure using AIPS among 22 European subpopulations. Only 952 known ancestry individuals were used in 22 subpopulations within Europe. The scores from PCA were first calculated then Inverse-Distance Weighted Interpolation without and with eigenvalue weight were applied to infer the ancestry membership. The number of admixture indicated the definition of admixture in AIPS.

**Supplementary Figure 4** Graphical Comparison of Population Structure using STRUCTURE and fastSTRUCTURE among 22 European subpopulations. The inferences of ancestry membership for 952 individuals were calculated by STRUCTURE and fastSTRUCTURE. (a) The prior population information was not given to compute inference of population membership using STRUCTURE. (b) To infer the population membership within 22 Europe countries, the prior population information was assigned in STRUCTURE. (c) fastSTRUCTURE was applied to infer 22 European subpopulations with simple model. (d) fastSTRUCTURE was used with logistic prior model.

**Supplementary Table 1** Distance-based clustering among 952 known and 3,426 unknown ancestry Europeans on 25,732 AIMs

| Population in 22 clusters | Population in 7 clusters | Sample Size | Centroid | | | | |
| --- | --- | --- | --- | --- | --- | --- | --- |
|  |  |  | Score1 | Score2 | Score3 | Score4 | Score5 |
| Adygei | S. European | 12 | 11591.92 | -2212.24 | -2367.38 | 3223.757 | 3178.474 |
| Ashkenazi Jewish American | Jews | 40 | 20169.72 | 3620.954 | 869.7979 | -161.391 | 60.11818 |
| Basque | Basque | 12 | 1263.031 | -2406.59 | 1362.048 | -1732.34 | -2806.74 |
| Bedouin | Arab | 22 | 22771.81 | -5744.59 | -2981.06 | 1286.952 | 4680.254 |
| CEPH Euro American | Great Britain | 21 | -4322.12 | 535.2313 | -215.943 | -528.366 | 128.0176 |
| Druze | Arab | 19 | 19996.68 | -5063.35 | -2398.69 | 851.0351 | 2978.709 |
| Dutch American | N. European | 3 | -3075.48 | 298.3933 | -380.633 | -869.586 | 236.0964 |
| Eastern Euro American | N. European | 11 | -2848.29 | 1565.323 | -3503.47 | 2265.713 | -1424.3 |
| German American | N. European | 16 | -2300.78 | 471.317 | -995.442 | -102.679 | -525.496 |
| Greek American | S. European | 7 | 12257.82 | -3692.28 | -1954.23 | 185.8735 | -361.976 |
| Hungarian American | N. European | 3 | -283.938 | -75.1753 | -2410.01 | 1138.751 | -745.296 |
| Irish | Great Britain | 84 | -4797.17 | -265.801 | 2563.482 | 1491.641 | 206.8548 |
| Italian American | S. European | 33 | 9261.946 | -3387.1 | -806.009 | -511.316 | -517.197 |
| Orcadian | Great Britain | 14 | -3996.66 | 203.2738 | 1032.663 | 190.5672 | 519.0328 |
| Palestinian | Arab | 21 | 21550.08 | -5498.04 | -2865.19 | 994.4572 | 3831.989 |
| Russian | Russian | 13 | -4478.12 | 3083.255 | -4533.56 | 3246.187 | -621.77 |
| Sardinian | S. European | 28 | 10934.98 | -5847.51 | 342.1359 | -4488.94 | -4881.06 |
| Scandinavian American | N. European | 6 | -5628.36 | 1615.751 | -1185.1 | -1006.6 | 892.1619 |
| Spanish | S. European | 12 | 5432.036 | -2537 | 148.0293 | -841.74 | -1186.6 |
| Swedish | N. European | 562 | -5946.41 | 1858.517 | -1849.93 | -843.028 | 788.9773 |
| Tuscan | S. European | 8 | 8677.799 | -2981.94 | -820.659 | -474.701 | -605.715 |
| United Kingdom American | Great Britain | 5 | -4176.13 | 241.1639 | 873.1527 | -124.392 | 401.243 |
| *Unknown** | *Unknown** | *3,424* | . | . | . | . | . |

*Note these samples from the New York Cancer Project and the Children’s Hospital of Philadelphia were self-identified as European.

**Supplementary Table 2** Rank-based on Closeness among 22 European subpopulations

| **POP** | **R1** | **R2** | **R3** | **R4** | **R5** | **R6** | **R7** | **R8** | **R9** | **R10** | **R11** | **R12** | **R13** | **R14** | **R15** | **R16** | **R17** | **R18** | **R19** | **R20** | **R21** | **R22** |
| --- | --- | --- | --- | --- | --- | --- | --- | --- | --- | --- | --- | --- | --- | --- | --- | --- | --- | --- | --- | --- | --- | --- |
| **ADYGEI** | ADYGEI | GRK | ITN | TUSC | SPAIN | DRUZE | PAL | AJA | BEDOUIN | SARD | HUN | BASQUE | GERM | UK | EEUR | NETH | ORCAD | CEU | RUS | IRISH | SCN | SWED |
| **AJA** | AJA | DRUZE | PAL | GRK | BEDOUIN | ADYGEI | ITN | TUSC | SARD | SPAIN | BASQUE | HUN | GERM | UK | NETH | EEUR | ORCAD | CEU | IRISH | RUS | SCN | SWED |
| **BASQUE** | BASQUE | SPAIN | UK | GERM | HUN | NETH | ORCAD | CEU | IRISH | TUSC | EEUR | ITN | SCN | SWED | SARD | RUS | GRK | ADYGEI | AJA | DRUZE | PAL | BEDOUIN |
| **BEDOUIN** | BEDOUIN | PAL | DRUZE | AJA | GRK | ADYGEI | ITN | TUSC | SARD | SPAIN | BASQUE | HUN | GERM | UK | NETH | EEUR | ORCAD | CEU | IRISH | RUS | SCN | SWED |
| **DRUZE** | DRUZE | PAL | BEDOUIN | GRK | ADYGEI | AJA | ITN | TUSC | SARD | SPAIN | BASQUE | HUN | GERM | UK | NETH | EEUR | ORCAD | CEU | IRISH | RUS | SCN | SWED |
| **PAL** | PAL | BEDOUIN | DRUZE | GRK | AJA | ADYGEI | ITN | TUSC | SARD | SPAIN | BASQUE | HUN | GERM | UK | NETH | EEUR | ORCAD | CEU | IRISH | RUS | SCN | SWED |
| **CEU** | CEU | NETH | ORCAD | UK | SCN | GERM | SWED | IRISH | EEUR | HUN | RUS | BASQUE | SPAIN | TUSC | ITN | ADYGEI | GRK | SARD | AJA | DRUZE | PAL | BEDOUIN |
| **EEUR** | EEUR | RUS | HUN | GERM | NETH | CEU | SWED | SCN | UK | ORCAD | IRISH | BASQUE | SPAIN | TUSC | ITN | ADYGEI | GRK | SARD | AJA | DRUZE | PAL | BEDOUIN |
| **GERM** | GERM | NETH | UK | CEU | HUN | ORCAD | EEUR | SCN | SWED | IRISH | BASQUE | RUS | SPAIN | TUSC | ITN | ADYGEI | GRK | SARD | AJA | DRUZE | PAL | BEDOUIN |
| **GRK** | GRK | ITN | TUSC | ADYGEI | SPAIN | SARD | DRUZE | PAL | AJA | BEDOUIN | BASQUE | HUN | GERM | UK | NETH | EEUR | ORCAD | CEU | IRISH | RUS | SCN | SWED |
| **HUN** | HUN | GERM | EEUR | NETH | UK | CEU | ORCAD | BASQUE | RUS | SCN | SWED | IRISH | SPAIN | TUSC | ITN | ADYGEI | GRK | SARD | AJA | DRUZE | PAL | BEDOUIN |
| **IRISH** | IRISH | ORCAD | UK | CEU | NETH | GERM | SCN | SWED | HUN | EEUR | BASQUE | RUS | SPAIN | TUSC | ITN | ADYGEI | GRK | SARD | AJA | DRUZE | PAL | BEDOUIN |
| **ITN** | ITN | TUSC | GRK | SPAIN | ADYGEI | SARD | BASQUE | HUN | DRUZE | GERM | UK | NETH | AJA | PAL | EEUR | ORCAD | CEU | BEDOUIN | IRISH | SCN | RUS | SWED |
| **TUSC** | TUSC | ITN | SPAIN | GRK | ADYGEI | SARD | BASQUE | HUN | GERM | UK | DRUZE | NETH | EEUR | ORCAD | AJA | CEU | PAL | IRISH | SCN | RUS | SWED | BEDOUIN |
| **NETH** | NETH | CEU | UK | GERM | ORCAD | SCN | SWED | HUN | IRISH | EEUR | BASQUE | RUS | SPAIN | TUSC | ITN | ADYGEI | GRK | SARD | AJA | DRUZE | PAL | BEDOUIN |
| **ORCAD** | ORCAD | UK | CEU | NETH | IRISH | GERM | SCN | SWED | HUN | EEUR | BASQUE | RUS | SPAIN | TUSC | ITN | ADYGEI | GRK | SARD | AJA | DRUZE | PAL | BEDOUIN |
| **RUS** | RUS | EEUR | SWED | SCN | GERM | HUN | CEU | NETH | ORCAD | UK | IRISH | BASQUE | SPAIN | TUSC | ITN | ADYGEI | GRK | SARD | AJA | DRUZE | PAL | BEDOUIN |
| **SARD** | SARD | ITN | TUSC | GRK | SPAIN | BASQUE | ADYGEI | DRUZE | HUN | AJA | PAL | GERM | UK | NETH | BEDOUIN | ORCAD | CEU | EEUR | IRISH | SCN | SWED | RUS |
| **SCN** | SCN | SWED | CEU | NETH | ORCAD | GERM | UK | IRISH | EEUR | RUS | HUN | BASQUE | SPAIN | TUSC | ITN | ADYGEI | GRK | SARD | AJA | DRUZE | PAL | BEDOUIN |
| **SWED** | SWED | SCN | CEU | NETH | ORCAD | GERM | UK | EEUR | RUS | IRISH | HUN | BASQUE | SPAIN | TUSC | ITN | ADYGEI | GRK | SARD | AJA | DRUZE | PAL | BEDOUIN |
| **SPAIN** | SPAIN | TUSC | ITN | BASQUE | HUN | GRK | SARD | GERM | UK | ADYGEI | NETH | ORCAD | CEU | EEUR | IRISH | SCN | SWED | RUS | DRUZE | AJA | PAL | BEDOUIN |
| **UK** | UK | ORCAD | NETH | CEU | GERM | IRISH | SCN | HUN | SWED | EEUR | BASQUE | RUS | SPAIN | TUSC | ITN | ADYGEI | GRK | SARD | AJA | DRUZE | PAL | BEDOUIN |

**Supplementary Table 3** Distance between two centroids among 22 European subpopulations

| **POP** | **ADYGEI** | **AJA** | **BASQUE** | **BEDOUIN** | **DRUZE** | **PAL** | **CEU** | **EEUR** | **GERM** | **GRK** | **HUN** | **IRISH** | **ITN** | **TUSC** | **NETH** | **ORCAD** | **RUS** | **SARD** | **SCN** | **SWED** | **SPAIN** | **UK** |
| --- | --- | --- | --- | --- | --- | --- | --- | --- | --- | --- | --- | --- | --- | --- | --- | --- | --- | --- | --- | --- | --- | --- |
| **ADYGEI** | 0.00 | 11800.87 | 13454.07 | 11993.97 | 9189.10 | 10752.09 | 16994.28 | 15689.89 | 15062.14 | 4956.20 | 12858.37 | 17564.90 | 6070.21 | 6282.39 | 15836.22 | 16633.08 | 17476.26 | 12058.67 | 18322.68 | 18619.12 | 8868.44 | 15556.99 |
| **AJA** | 11800.87 | 0.00 | 20117.85 | 11522.03 | 9781.58 | 10703.65 | 24712.12 | 23691.21 | 22774.33 | 11151.41 | 21097.63 | 25378.63 | 13090.32 | 13381.39 | 23526.04 | 24414.25 | 25477.04 | 14776.80 | 25984.54 | 26335.36 | 15951.87 | 23279.15 |
| **BASQUE** | 13454.07 | 20117.85 | 0.00 | 23617.98 | 20305.55 | 22147.06 | 7239.01 | 8616.91 | 5865.34 | 11966.30 | 5878.26 | 7888.68 | 8739.60 | 8154.88 | 6259.62 | 7024.77 | 11287.89 | 10877.80 | 9184.89 | 9706.03 | 4819.19 | 5741.88 |
| **BEDOUIN** | 11993.97 | 11522.03 | 23617.98 | 0.00 | 3403.87 | 1540.47 | 28375.61 | 27355.38 | 26462.18 | 11935.22 | 24361.65 | 28997.60 | 14934.84 | 15555.87 | 27124.90 | 28045.49 | 29237.89 | 16611.27 | 29725.43 | 30058.26 | 18865.51 | 26847.97 |
| **DRUZE** | 9189.10 | 9781.58 | 20305.55 | 3403.87 | 0.00 | 1888.19 | 25249.68 | 24257.90 | 23301.89 | 8577.81 | 21216.54 | 25893.55 | 11604.52 | 12229.21 | 23992.39 | 24933.54 | 26242.15 | 13436.66 | 26656.24 | 26998.68 | 15555.41 | 23707.84 |
| **PAL** | 10752.09 | 10703.65 | 22147.06 | 1540.47 | 1888.19 | 0.00 | 26996.55 | 25977.14 | 25063.45 | 10424.80 | 22963.01 | 27647.86 | 13448.71 | 14073.04 | 25740.70 | 26682.24 | 27906.84 | 15134.50 | 28368.18 | 28702.48 | 17396.06 | 25468.21 |
| **CEU** | 16994.28 | 24712.12 | 7239.01 | 28375.61 | 25249.68 | 26996.55 | 0.00 | 4925.19 | 2303.38 | 17220.16 | 5003.50 | 3560.84 | 14165.98 | 13500.86 | 1328.64 | 1563.65 | 6321.80 | 17737.13 | 2150.41 | 2755.92 | 10443.93 | 1984.02 |
| **EEUR** | 15689.89 | 23691.21 | 8616.91 | 27355.38 | 24257.90 | 25977.14 | 4925.19 | 0.00 | 3768.87 | 16238.48 | 3491.97 | 6871.64 | 13674.60 | 12996.33 | 4898.59 | 5642.14 | 2761.46 | 17812.67 | 5401.91 | 5194.38 | 10497.20 | 5553.94 |
| **GERM** | 15062.14 | 22774.33 | 5865.34 | 26462.18 | 23301.89 | 25063.45 | 2303.38 | 3768.87 | 0.00 | 15176.18 | 2820.89 | 4745.47 | 12197.85 | 11516.49 | 1475.25 | 2870.26 | 5941.71 | 15972.17 | 3904.39 | 4268.64 | 8547.77 | 2115.48 |
| **GRK** | 4956.20 | 11151.41 | 11966.30 | 11935.22 | 8577.81 | 10424.80 | 17220.16 | 16238.48 | 15176.18 | 0.00 | 13101.13 | 18029.10 | 3300.92 | 3886.04 | 15968.14 | 17002.28 | 18495.50 | 7344.32 | 18753.18 | 19094.45 | 7238.55 | 15711.33 |
| **HUN** | 12858.37 | 21097.63 | 5878.26 | 24361.65 | 21216.54 | 22963.01 | 5003.50 | 3491.97 | 2820.89 | 13101.13 | 0.00 | 6795.07 | 10365.34 | 9690.74 | 4128.86 | 5311.45 | 6044.12 | 14681.09 | 6340.95 | 6511.36 | 7118.64 | 4422.25 |
| **IRISH** | 17564.90 | 25378.63 | 7888.68 | 28997.60 | 25893.55 | 27647.86 | 3560.84 | 6871.64 | 4745.47 | 18029.10 | 6795.07 | 0.00 | 14942.91 | 14315.39 | 4186.46 | 2234.95 | 8090.14 | 18580.75 | 4999.26 | 5576.87 | 11207.97 | 3226.06 |
| **ITN** | 6070.21 | 13090.32 | 8739.60 | 14934.84 | 11604.52 | 13448.71 | 14165.98 | 13674.60 | 12197.85 | 3300.92 | 10365.34 | 14942.91 | 0.00 | 717.52 | 12910.15 | 13915.03 | 16083.47 | 6710.80 | 15783.80 | 16177.65 | 3995.36 | 12595.41 |
| **TUSC** | 6282.39 | 13381.39 | 8154.88 | 15555.87 | 12229.21 | 14073.04 | 13500.86 | 12996.33 | 11516.49 | 3886.04 | 9690.74 | 14315.39 | 717.52 | 0.00 | 12245.73 | 13263.81 | 15410.84 | 7003.53 | 15115.11 | 15506.04 | 3374.18 | 11943.76 |
| **NETH** | 15836.22 | 23526.04 | 6259.62 | 27124.90 | 23992.39 | 25740.70 | 1328.64 | 4898.59 | 1475.25 | 15968.14 | 4128.86 | 4186.46 | 12910.15 | 12245.73 | 0.00 | 2014.63 | 6681.52 | 16549.11 | 3057.47 | 3625.11 | 9218.57 | 1415.30 |
| **ORCAD** | 16633.08 | 24414.25 | 7024.77 | 28045.49 | 24933.54 | 26682.24 | 1563.65 | 5642.14 | 2870.26 | 17002.28 | 5311.45 | 2234.95 | 13915.03 | 13263.81 | 2014.63 | 0.00 | 7081.34 | 17638.12 | 3338.79 | 3998.93 | 10181.19 | 1412.14 |
| **RUS** | 17476.26 | 25477.04 | 11287.89 | 29237.89 | 26242.15 | 27906.84 | 6321.80 | 2761.46 | 5941.71 | 18495.50 | 6044.12 | 8090.14 | 16083.47 | 15410.84 | 6681.52 | 7081.34 | 0.00 | 20470.96 | 5921.78 | 5437.59 | 13073.49 | 7430.14 |
| **SARD** | 12058.67 | 14776.80 | 10877.80 | 16611.27 | 13436.66 | 15134.50 | 17737.13 | 17812.67 | 15972.17 | 7344.32 | 14681.09 | 18580.75 | 6710.80 | 7003.53 | 16549.11 | 17638.12 | 20470.96 | 0.00 | 19437.66 | 19864.80 | 8165.72 | 16256.93 |
| **SCN** | 18322.68 | 25984.54 | 9184.89 | 29725.43 | 26656.24 | 28368.18 | 2150.41 | 5401.91 | 3904.39 | 18753.18 | 6340.95 | 4999.26 | 15783.80 | 15115.11 | 3057.47 | 3338.79 | 5921.78 | 19437.66 | 0.00 | 799.83 | 12192.59 | 4015.94 |
| **SWED** | 18619.12 | 26335.36 | 9706.03 | 30058.26 | 26998.68 | 28702.48 | 2755.92 | 5194.38 | 4268.64 | 19094.45 | 6511.36 | 5576.87 | 16177.65 | 15506.04 | 3625.11 | 3998.93 | 5437.59 | 19864.80 | 799.83 | 0.00 | 12636.41 | 4658.84 |
| **SPAIN** | 8868.44 | 15951.87 | 4819.19 | 18865.51 | 15555.41 | 17396.06 | 10443.93 | 10497.20 | 8547.77 | 7238.55 | 7118.64 | 11207.97 | 3995.36 | 3374.18 | 9218.57 | 10181.19 | 13073.49 | 8165.72 | 12192.59 | 12636.41 | 0.00 | 8833.55 |
| **UK** | 15556.99 | 23279.15 | 5741.88 | 26847.97 | 23707.84 | 25468.21 | 1984.02 | 5553.94 | 2115.48 | 15711.33 | 4422.25 | 3226.06 | 12595.41 | 11943.76 | 1415.30 | 1412.14 | 7430.14 | 16256.93 | 4015.94 | 4658.84 | 8833.55 | 0.00 |

**Supplementary Table 4** The Average Percent of Correctly Inferred Proportions from AIPS and ADMIXTURE without Population Information

| AIPS | ADYGEI | AJA_NS | BASQUE | BEDOUIN | DRUZE | PAL | CEU | EEUR | GERM | GRK | HUN | IRISH | ITN | TUSC | NETH | ORCAD | RUS | SARD | SCN | SWED | SPAIN | UK |
| --- | --- | --- | --- | --- | --- | --- | --- | --- | --- | --- | --- | --- | --- | --- | --- | --- | --- | --- | --- | --- | --- | --- |
| ADYGEI | **0.81** | 0.00 | 0.00 | 0.00 | 0.00 | 0.00 | 0.00 | 0.00 | 0.00 | 0.10 | 0.00 | 0.00 | 0.05 | 0.03 | 0.00 | 0.00 | 0.00 | 0.00 | 0.00 | 0.00 | 0.00 | 0.00 |
| AJA_NS | 0.00 | **0.89** | 0.00 | 0.00 | 0.04 | 0.02 | 0.00 | 0.00 | 0.00 | 0.05 | 0.00 | 0.00 | 0.00 | 0.00 | 0.00 | 0.00 | 0.00 | 0.00 | 0.00 | 0.00 | 0.00 | 0.00 |
| BASQUE | 0.00 | 0.00 | **0.82** | 0.00 | 0.00 | 0.00 | 0.00 | 0.00 | 0.03 | 0.00 | 0.04 | 0.00 | 0.00 | 0.00 | 0.00 | 0.00 | 0.00 | 0.00 | 0.00 | 0.00 | 0.11 | 0.00 |
| BEDOUIN | 0.00 | 0.00 | 0.00 | **0.76** | 0.09 | 0.14 | 0.00 | 0.00 | 0.00 | 0.00 | 0.00 | 0.00 | 0.00 | 0.00 | 0.00 | 0.00 | 0.00 | 0.00 | 0.00 | 0.00 | 0.00 | 0.00 |
| DRUZE | 0.00 | 0.00 | 0.00 | 0.04 | **0.78** | 0.17 | 0.00 | 0.00 | 0.00 | 0.02 | 0.00 | 0.00 | 0.00 | 0.00 | 0.00 | 0.00 | 0.00 | 0.00 | 0.00 | 0.00 | 0.00 | 0.00 |
| PAL | 0.00 | 0.00 | 0.00 | 0.14 | 0.14 | **0.72** | 0.00 | 0.00 | 0.00 | 0.00 | 0.00 | 0.00 | 0.00 | 0.00 | 0.00 | 0.00 | 0.00 | 0.00 | 0.00 | 0.00 | 0.00 | 0.00 |
| CEU | 0.00 | 0.00 | 0.00 | 0.00 | 0.00 | 0.00 | **0.34** | 0.00 | 0.10 | 0.00 | 0.00 | 0.00 | 0.00 | 0.00 | 0.17 | 0.02 | 0.00 | 0.00 | 0.11 | 0.09 | 0.00 | 0.19 |
| EEUR | 0.00 | 0.00 | 0.00 | 0.00 | 0.00 | 0.00 | 0.02 | **0.58** | 0.03 | 0.00 | 0.17 | 0.00 | 0.00 | 0.00 | 0.00 | 0.00 | 0.18 | 0.00 | 0.00 | 0.02 | 0.00 | 0.00 |
| GERM | 0.00 | 0.00 | 0.03 | 0.00 | 0.00 | 0.00 | 0.15 | 0.05 | **0.51** | 0.00 | 0.12 | 0.00 | 0.00 | 0.00 | 0.11 | 0.00 | 0.00 | 0.00 | 0.00 | 0.00 | 0.00 | 0.02 |
| GRK | 0.00 | 0.00 | 0.00 | 0.00 | 0.19 | 0.00 | 0.00 | 0.00 | 0.00 | **0.43** | 0.00 | 0.00 | 0.20 | 0.18 | 0.00 | 0.00 | 0.00 | 0.00 | 0.00 | 0.00 | 0.00 | 0.00 |
| HUN | 0.00 | 0.00 | 0.00 | 0.00 | 0.00 | 0.00 | 0.00 | 0.16 | 0.10 | 0.00 | **0.66** | 0.00 | 0.00 | 0.00 | 0.08 | 0.00 | 0.00 | 0.00 | 0.00 | 0.00 | 0.00 | 0.00 |
| IRISH | 0.00 | 0.00 | 0.00 | 0.00 | 0.00 | 0.00 | 0.00 | 0.00 | 0.00 | 0.00 | 0.00 | **0.83** | 0.00 | 0.00 | 0.00 | 0.08 | 0.00 | 0.00 | 0.00 | 0.00 | 0.00 | 0.09 |
| ITN | 0.00 | 0.00 | 0.01 | 0.00 | 0.02 | 0.00 | 0.00 | 0.00 | 0.00 | 0.33 | 0.00 | 0.00 | **0.18** | 0.20 | 0.00 | 0.03 | 0.00 | 0.00 | 0.00 | 0.00 | 0.24 | 0.00 |
| TUSC | 0.00 | 0.00 | 0.00 | 0.00 | 0.00 | 0.00 | 0.00 | 0.00 | 0.00 | 0.07 | 0.00 | 0.00 | 0.21 | **0.60** | 0.00 | 0.00 | 0.00 | 0.00 | 0.00 | 0.00 | 0.11 | 0.00 |
| NETH | 0.00 | 0.00 | 0.00 | 0.00 | 0.00 | 0.00 | 0.33 | 0.00 | 0.00 | 0.00 | 0.00 | 0.00 | 0.00 | 0.00 | **0.67** | 0.00 | 0.00 | 0.00 | 0.00 | 0.00 | 0.00 | 0.00 |
| ORCAD | 0.00 | 0.00 | 0.00 | 0.00 | 0.00 | 0.00 | 0.02 | 0.00 | 0.00 | 0.00 | 0.00 | 0.03 | 0.00 | 0.00 | 0.09 | **0.66** | 0.00 | 0.00 | 0.00 | 0.00 | 0.00 | 0.21 |
| RUS | 0.00 | 0.00 | 0.00 | 0.00 | 0.00 | 0.00 | 0.00 | 0.10 | 0.00 | 0.00 | 0.04 | 0.00 | 0.00 | 0.00 | 0.00 | 0.00 | **0.81** | 0.00 | 0.00 | 0.05 | 0.00 | 0.00 |
| SARD | 0.00 | 0.00 | 0.00 | 0.00 | 0.00 | 0.00 | 0.00 | 0.00 | 0.00 | 0.02 | 0.00 | 0.00 | 0.07 | 0.04 | 0.00 | 0.00 | 0.00 | **0.87** | 0.00 | 0.00 | 0.00 | 0.00 |
| SCN | 0.00 | 0.00 | 0.00 | 0.00 | 0.00 | 0.00 | 0.17 | 0.00 | 0.00 | 0.00 | 0.00 | 0.00 | 0.00 | 0.00 | 0.00 | 0.00 | 0.00 | 0.00 | **0.67** | 0.17 | 0.00 | 0.00 |
| SWED | 0.00 | 0.00 | 0.00 | 0.00 | 0.00 | 0.00 | 0.09 | 0.00 | 0.01 | 0.00 | 0.00 | 0.00 | 0.00 | 0.00 | 0.00 | 0.00 | 0.01 | 0.00 | 0.30 | **0.59** | 0.00 | 0.00 |
| SPAIN | 0.00 | 0.00 | 0.16 | 0.00 | 0.00 | 0.00 | 0.00 | 0.00 | 0.00 | 0.00 | 0.02 | 0.00 | 0.01 | 0.22 | 0.00 | 0.00 | 0.00 | 0.00 | 0.00 | 0.00 | **0.60** | 0.00 |
| UK | 0.00 | 0.00 | 0.00 | 0.00 | 0.00 | 0.00 | 0.09 | 0.00 | 0.00 | 0.00 | 0.00 | 0.08 | 0.00 | 0.00 | 0.10 | 0.17 | 0.00 | 0.00 | 0.00 | 0.00 | 0.00 | **0.56** |
| UNKNOWN | 0.00 | 0.09 | 0.03 | 0.00 | 0.01 | 0.00 | 0.04 | 0.02 | 0.14 | 0.05 | 0.09 | 0.08 | 0.02 | 0.04 | 0.11 | 0.08 | 0.00 | 0.00 | 0.01 | 0.00 | 0.09 | 0.09 |

| ADMIXTURE | POP1 | POP2 | POP3 | POP4 | POP5 | POP6 | POP7 | POP8 | POP9 | POP10 | POP11 | POP12 | POP13 | POP14 | POP15 | POP16 | POP17 | POP18 | POP19 | POP20 | POP21 | POP22 |
| --- | --- | --- | --- | --- | --- | --- | --- | --- | --- | --- | --- | --- | --- | --- | --- | --- | --- | --- | --- | --- | --- | --- |
| ADYGEI | 0.03 | **0.30** | 0.10 | 0.02 | 0.02 | 0.02 | 0.00 | 0.01 | 0.12 | 0.01 | 0.04 | 0.07 | 0.06 | 0.02 | 0.02 | 0.01 | 0.01 | 0.01 | 0.01 | 0.03 | 0.07 | 0.03 |
| AJA_NS | 0.01 | 0.03 | 0.03 | 0.03 | 0.02 | **0.43** | 0.02 | 0.04 | 0.04 | 0.04 | 0.02 | 0.01 | 0.03 | 0.02 | 0.04 | 0.03 | 0.03 | 0.03 | 0.02 | 0.04 | 0.01 | 0.02 |
| BASQUE | 0.00 | 0.00 | 0.01 | 0.01 | 0.01 | 0.01 | **0.21** | 0.01 | 0.01 | 0.07 | 0.07 | 0.04 | 0.01 | 0.13 | 0.02 | 0.04 | 0.01 | 0.13 | 0.05 | 0.01 | 0.01 | 0.15 |
| BEDOUIN | 0.02 | 0.29 | 0.00 | 0.01 | **0.33** | 0.03 | 0.04 | 0.01 | 0.00 | 0.01 | 0.01 | 0.02 | 0.08 | 0.01 | 0.03 | 0.01 | 0.01 | 0.02 | 0.01 | 0.05 | 0.00 | 0.02 |
| DRUZE | 0.01 | 0.24 | 0.03 | 0.02 | 0.08 | 0.04 | 0.06 | 0.02 | 0.04 | 0.01 | 0.01 | 0.01 | **0.29** | 0.03 | 0.02 | 0.00 | 0.01 | 0.02 | 0.01 | 0.02 | 0.00 | 0.01 |
| PAL | 0.02 | **0.37** | 0.02 | 0.03 | 0.13 | 0.03 | 0.05 | 0.02 | 0.02 | 0.02 | 0.03 | 0.02 | 0.07 | 0.02 | 0.01 | 0.03 | 0.04 | 0.02 | 0.01 | 0.03 | 0.01 | 0.02 |
| CEU | **0.09** | 0.02 | 0.05 | 0.07 | 0.01 | 0.01 | 0.02 | 0.05 | 0.04 | 0.07 | 0.06 | 0.04 | 0.02 | 0.05 | 0.05 | 0.06 | 0.05 | 0.04 | 0.06 | 0.05 | 0.03 | 0.06 |
| EEUR | 0.04 | 0.03 | 0.04 | 0.02 | 0.02 | 0.02 | 0.03 | 0.04 | 0.04 | 0.06 | 0.03 | 0.03 | 0.03 | 0.02 | 0.04 | 0.05 | 0.05 | 0.07 | 0.04 | 0.03 | **0.25** | 0.02 |
| GERM | 0.05 | 0.02 | 0.04 | 0.04 | 0.02 | 0.02 | 0.03 | 0.06 | 0.05 | 0.04 | 0.05 | 0.02 | 0.02 | 0.06 | 0.06 | 0.05 | 0.05 | 0.05 | 0.07 | 0.07 | **0.08** | 0.04 |
| GRK | 0.01 | **0.20** | 0.03 | 0.04 | 0.03 | 0.03 | 0.15 | 0.07 | 0.07 | 0.05 | 0.02 | 0.01 | 0.05 | 0.02 | 0.01 | 0.02 | 0.03 | 0.03 | 0.04 | 0.03 | 0.04 | 0.02 |
| HUN | 0.04 | 0.06 | 0.02 | 0.10 | 0.05 | 0.01 | 0.04 | 0.06 | 0.01 | 0.04 | 0.05 | 0.03 | 0.03 | 0.04 | 0.03 | 0.03 | 0.05 | 0.03 | 0.03 | 0.04 | **0.15** | 0.05 |
| IRISH | 0.01 | 0.01 | 0.05 | 0.05 | 0.02 | 0.01 | 0.01 | 0.05 | 0.04 | 0.05 | 0.07 | **0.19** | 0.02 | 0.04 | 0.06 | 0.05 | 0.05 | 0.05 | 0.04 | 0.04 | 0.02 | 0.05 |
| ITN | 0.01 | 0.14 | 0.04 | 0.03 | 0.04 | 0.02 | **0.15** | 0.04 | 0.05 | 0.04 | 0.03 | 0.01 | 0.04 | 0.04 | 0.03 | 0.05 | 0.04 | 0.05 | 0.03 | 0.03 | 0.02 | 0.05 |
| TUSC | 0.01 | 0.13 | 0.03 | 0.01 | 0.04 | 0.03 | **0.14** | 0.05 | 0.03 | 0.05 | 0.04 | 0.01 | 0.04 | 0.06 | 0.04 | 0.06 | 0.05 | 0.04 | 0.04 | 0.03 | 0.03 | 0.06 |
| NETH | 0.07 | 0.04 | 0.08 | 0.05 | 0.01 | 0.01 | 0.02 | 0.07 | 0.03 | 0.07 | 0.02 | 0.02 | 0.01 | 0.04 | 0.07 | 0.03 | 0.01 | 0.07 | 0.09 | **0.12** | 0.02 | 0.05 |
| ORCAD | 0.07 | 0.02 | 0.04 | 0.04 | 0.02 | 0.01 | 0.01 | 0.03 | 0.04 | 0.03 | 0.04 | **0.11** | 0.02 | 0.08 | 0.04 | 0.04 | 0.07 | 0.07 | 0.03 | 0.09 | 0.01 | 0.07 |
| RUS | 0.08 | 0.03 | 0.05 | 0.03 | 0.02 | 0.02 | 0.00 | 0.01 | 0.04 | 0.05 | 0.04 | 0.03 | 0.02 | 0.05 | 0.04 | 0.03 | 0.04 | 0.03 | 0.04 | 0.02 | **0.31** | 0.01 |
| SARD | 0.02 | 0.01 | 0.02 | 0.02 | 0.02 | 0.01 | **0.56** | 0.03 | 0.01 | 0.02 | 0.01 | 0.02 | 0.02 | 0.02 | 0.02 | 0.03 | 0.04 | 0.02 | 0.02 | 0.05 | 0.00 | 0.03 |
| SCN | **0.17** | 0.01 | 0.08 | 0.04 | 0.03 | 0.01 | 0.02 | 0.05 | 0.05 | 0.05 | 0.05 | 0.03 | 0.01 | 0.05 | 0.06 | 0.05 | 0.01 | 0.06 | 0.05 | 0.04 | 0.03 | 0.06 |
| SWED | **0.18** | 0.01 | 0.05 | 0.05 | 0.02 | 0.01 | 0.02 | 0.05 | 0.04 | 0.05 | 0.05 | 0.02 | 0.02 | 0.04 | 0.05 | 0.04 | 0.04 | 0.04 | 0.06 | 0.05 | 0.05 | 0.04 |
| SPAIN | 0.01 | 0.06 | 0.04 | 0.03 | 0.04 | 0.03 | **0.17** | 0.01 | 0.05 | 0.05 | 0.05 | 0.03 | 0.04 | 0.06 | 0.04 | 0.04 | 0.06 | 0.05 | 0.03 | 0.04 | 0.02 | 0.07 |
| UK | 0.07 | 0.03 | 0.06 | 0.04 | 0.02 | 0.01 | 0.03 | 0.06 | 0.04 | 0.03 | **0.09** | **0.09** | 0.01 | 0.02 | 0.04 | 0.07 | 0.06 | 0.04 | 0.05 | 0.06 | 0.02 | 0.05 |
| UNKNOWN | 0.04 | 0.05 | 0.05 | 0.05 | 0.03 | 0.07 | 0.05 | 0.05 | 0.04 | 0.05 | 0.05 | 0.06 | 0.03 | 0.05 | 0.05 | 0.05 | 0.05 | 0.05 | 0.05 | 0.04 | 0.04 | 0.04 |
